# Removal of hexavalent chromium from contaminated synthetic groundwater via functionalized carbon nanomaterials modified with zinc and potassium

**DOI:** 10.1038/s41598-025-01025-y

**Published:** 2025-09-30

**Authors:** Peter D. Ibikunle, Olugbenga O. Elemile, Praise O. Ejigboye, David O. Bala, Ayodeji P. Olawolu, Asegun A. Adebayo, Opeyemi S. Olajide

**Affiliations:** 1 Department of Civil Engineering, Landmark University, Omu-Aran, Nigeria; 2 Department of Civil Engineering, Olusegun Agagu University of Science and Technology, Okitipupa, Nigeria

**Keywords:** Carbon nanotubes, Chromium(VI), Adsorption, Water treatment, Graphitic carbon, Environmental sciences, Engineering

## Abstract

Chromium has been shown to be a significant contributor to water pollution, leading to cancer. This study aimed to investigate the potential of various functionalized carbon nanomaterials for removing Cr(VI) from synthetic groundwater. Functionalized carbon nanomaterials with layered and tube-like structures were designed on the basis of thermal methods (KOH-activated micrographite sheets) and impregnation methods by anchoring K and Zn on carbon nanotubes (CNTs), respectively, for the removal of Cr(VI) from contaminated synthetic groundwater. Several experimental parameters, including the concentration of the solution, pH, adsorbent dosage, and duration of contact, were systematically varied in a series of batch experiments. The optimal conditions were determined to be a 30 mg/20 mL adsorbent dosage (after the dosage test was 5–50 mg), a pH of 2 (tested at 1–240 min), and a constant agitation speed for all adsorbents. The maximum Cr(VI) removal by K-CNTs (83.04%) occurred at pH 4 and 25 °C and at a constant agitation speed. Zn-CNTs achieved up to 79% removal efficiency at pH 2. However, at neutral pH (8), only K-CNTs maintained a high removal capacity (97%), whereas the other adsorbents exhibited an approximately 53% decrease in removal efficiency. The Cr(VI) removal efficiency reached 95% and 32.7% after five adsorption/desorption cycle tests for K and Zn-CNTs, respectively, at pH = 8, and K-CNTs achieved 91% removal after six cycles, which is particularly noteworthy. Fourier transform infrared (FTIR) spectroscopy was employed to observe the adsorption processes in the polluted water samples, both before and after treatment. The results indicated an increase in the specific surface area and beneficial development of adsorption sites on the CNTs. To elucidate the adsorption mechanisms, the experimental data were analyzed via various isotherm models, namely, the Langmuir, Freundlich, Temkin, and Sips models. Additionally, the adsorption kinetics were examined via pseudo-first-order and pseudo-second-order kinetic models to characterize the temporal aspects of the adsorption process.

**Supplementary Information:**

The online version contains supplementary material available at 10.1038/s41598-025-01025-y.

## Introduction

Through direct and indirect pollution, human activities impact groundwater^1^, and all these activities contribute to groundwater pollution. These activities include open dump sites, home sewage, effluent runoff from industries, and fertilizer application, all of which can result in health and environmental problems. The World Water Council Project predicts that since the world’s population has tripled in the last century, approximately 3.9 billion people will stay in the “water scarce” region^2^. Sicknesses from this tainted water have taken up 2.2 million people every year, and approximately 1.1 billion people still have no access to pure water^3,4^.

According to^5^, a significant issue has arisen because several unhygienic practices have caused heavy metal contamination and groundwater pollution. Therefore, there is a need for more effective water treatment technologies. Research worldwide shows that heavy metals from different sources in drinking water are harmful and are becoming a serious threat to human health^6–8^.

Chromium, a heavy metal in water, is usually utilized in the copying and tinting, electroplating, leathering, jet streaming, corrosion protection, and painting industries. The two fundamental sources of commercial Cr are trivalent Cr(III) and hexavalent Cr(VI)^9^. When adsorbents are added to alkaline or normal environments, Cr(III), a low-hazard pollutant, can be adsorbed more easily than other Cr species^10,11^. In addition, the hexavalent form of Cr is more hazardous than the trivalent form. According to previous reports, hexavalent chromium can cause cancer and mutations in both humans and animals. It causes perforation of the nasal septum, dermatitis, and bronchitis^12,13^. Moreover, contamination by this herb is recognized as a major environmental problem that has been found to be harmful to drinking water. Numerous epidemiological studies have shown that chronic exposure to Cr(VI) causes several illnesses, including fibrosis, cancer, and respiratory problems^14^.

Unlike Cr(VI), Cr(III) can be removed without difficulty by discharging insoluble Cr(OH)_3_ because of its nontoxic and inert nature^15^. For example, manufactured 3D Ni@N-C materials doped with NiO nanoparticles and unified integrated CNTs were used for the removal of Cr(III). Ni@N-C showed better stimulant performance for the catalytic attrition of Cr(VI) to nontoxic Cr(III) when an acrid was used as the reducing agent^16^. To date, several catalysts for the attrition of Cr(VI) by formic acid include transition metals (such as Co, Fe, and Ni) and noble metals with admixtures (Pd-Cu, Au-Pd, Ni-Mo, and Pd-Cd)^17,18^. Adsorbents, including activated carbons^19,20^, carbon nanotubes (CNTs)^21^, inorganic and organic composite materials^22–25^, graphene oxides^26,27^, carbon nanofibers (CNFs)^28^, and metal‒organic frameworks (MOFs)^28–30^, have been frequently studied for the removal of Cr(VI) from aqueous solutions. However, adsorbents made of MOFs, graphene oxides, and various composite materials typically have problems with stability at low pH values or difficulties in large-scale manufacturing. In addition, graphitic carbon nitride (g-C_3_N_4_), an organic nonmetal semiconductor, has various benefits, making it a good adsorbent^31^.

While examining particular heavy metals (HMs) and the dangers they pose in groundwater samples in southwest Nigeria, ^32^ reported that Pb, Cd, Fe, and Zn were present at levels above the recommended limits. Additionally, ^33^ noted that the principal water sources for Omu-Aran, a city located inside the southern boundary of Nigeria’s tropical Savanah zone, were hand-dug shallow wells and boreholes. Most of these hand-dug shallow wells are either outdated or in need of repair because of their age, lack of effectiveness, or both. Seven heavy metals, including lead (Pb), aluminum (Al), iron (Fe), manganese (Mn), chromium (Cr), copper (Cu), and zinc (Zn), were examined^33^. The results suggest that the average Cr concentration is 0.153 ± 0.175 mg/L, which exceeds the WHO (2017) safety guidelines. The maximum allowable Cr(VI) concentrations in drinking water and wastewater are 20 µg L^−1^ and 200 µg L^−1^, respectively, according to the US Environmental Protection Agency (US EPA) standards^34^.

Water treatment is therefore critical because it aids in the removal of pollutants and harmful compounds from water, rendering it safe to drink and use for other purposes^35^. Given that the use of an adsorption treatment method with impregnated CNTs for chromium obliteration from drinking water is still lacking in the literature, methods that can eliminate/deplete this contaminant Cr(VI) would be highly favorable. According to ^33^, the lifetime carcinogenic risk (LTCR) estimate for Cr vulnerability in infants ranged from 0 to 2.14 × 10^−4^, indicating that the current Cr intensity in Omu–Aran hand-dug wells is dangerous, particularly for children.

This study aimed to investigate the potential of functionalized carbon nanomaterials for removing Cr(VI) from contaminated synthetic groundwater to achieve water purification and adsorbent recycling. The objectives of this study include synthesizing and characterizing the physical and chemical properties of novel functionalized carbon nanotubes and comparing the performance of conventional activated carbon with that of novel functionalized carbon nanotubes. Finally, chromium adsorption from water under various experimental conditions was investigated, and the effects of other operating parameters were evaluated.

## Materials and methods

Graphite powder (99.95% metal basis, ≥ 44 μm) and short multiwalled carbon nanotubes (CNTs) (95% metal basis, thickness 30–50 nm, length 0.5–2 μm) were obtained from the Institute of Urban Environment, Chinese Academy of Sciences, Xiamen, China, and were used as received to prepare activated graphitic carbon and functionalized CNTs. Potassium hydroxide (KOH) (M, 0.02% K) and zinc acetate (ZnAC_2_) were obtained from Landmark University’s Industrial Chemistry Department for use in the impregnation/functionalization of carbon nanotubes.

Potassium dichromate (K_2_Cr_2_O_7_) (Scharlau, Extra Pure) was obtained from the Biochemistry Department of Landmark University. Diphenylcarbazide (Merck) was purchased from the Central Research and Diagnostic Laboratory (Ilorin, Nigeria). Sodium hydroxide (NaOH) (M, 0.02% K), concentrated HCl (Scharlau, 38% impurity), 1% H_3_PO_4,_ acetone, concentrated H_2_SO_4_ and deionized water were obtained from the Industrial Chemistry and Biochemistry Department of Landmark University.

### Synthesis of activated graphitic carbon

The as-received graphite powder was used to prepare activated graphitic carbon. Typically, 1 g of graphite was dissolved in 30 g of water to yield a 3-weight% dispersion. After 30 min of stirring, 1 g of KOH powder was added to the graphite solution, and the mixture was stirred for 120 min at room temperature. The prepared graphite/KOH solution was then placed in an air-circulating oven at 80 °C to allow the water to evaporate. Following evaporation, the resulting powder was heated in a furnace at 800 °C for 120 min. The produced powder was subsequently repeatedly rinsed with 1 M HCl and distilled water to eliminate any remaining KOH. The resulting powder was dried overnight at 80 °C in an oven under air circulation. This process yielded a blackish synthesized graphitic carbon powder, which was subsequently ground in a mortar to obtain the final powder. In conclusion, the finished item (designated AC-1) and as-received graphite powder (designated AC-0) were gathered and dried in an oven.

### Preparation of functionalized CNTs

Previous studies on adsorbent modification revealed that impregnation at 7 wt% resulted in the best performance when 0.35 kg of graphitic carbon was used as the impregnation material, although this was only valid for selected impregnation materials^36,37^.

**Fig. 1:**
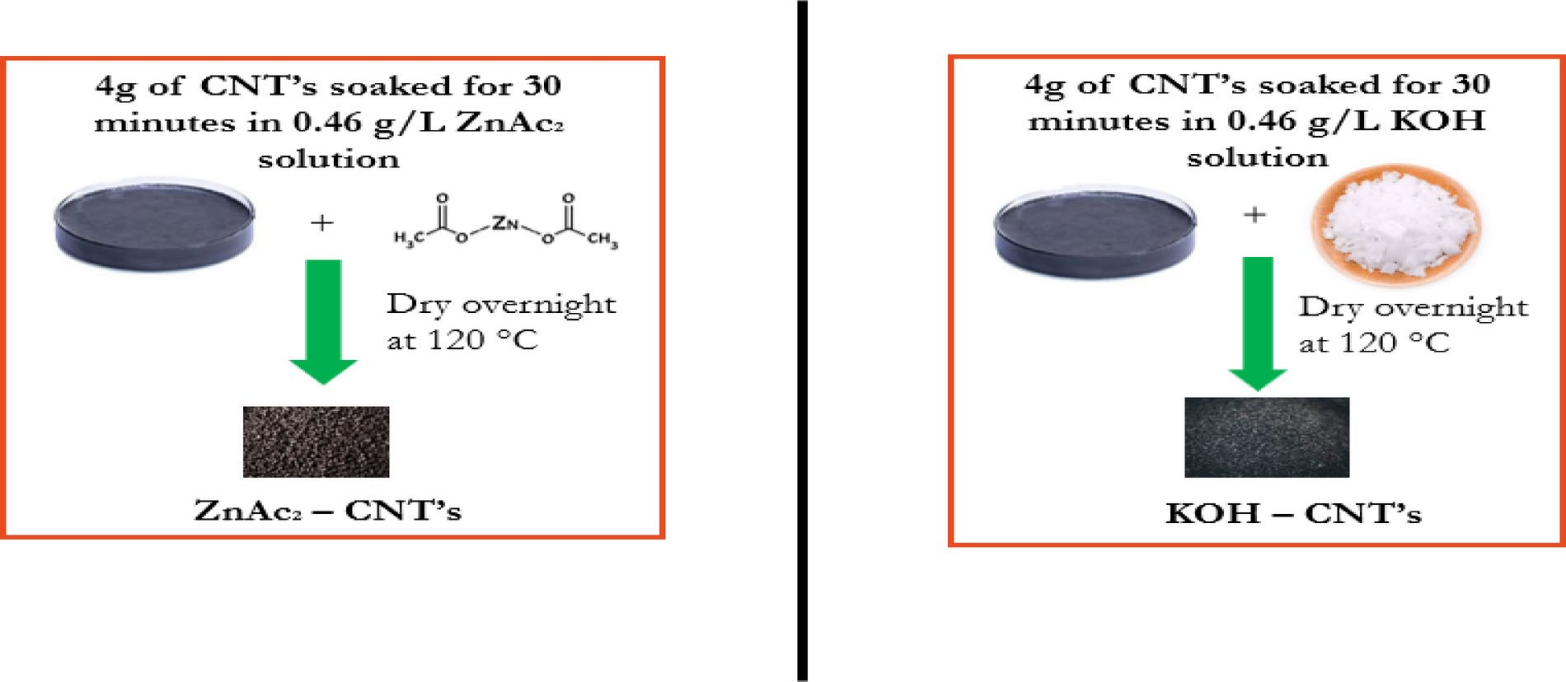
Procedures for CNT modification.

Unlike graphitic carbon, CNTs exhibit excellent mechanical, chemical, optical, and electronic properties. Thus, in this study, 4 g of CNTs was used as the impregnation material and modified with KOH or Zn acetate (ZnAC_2_) at a constant impregnation ratio. The CNTs were dried overnight at 120 °C and then soaked for 30 min in 0.46 g/L impregnated solution. The terms KOH–CNT (K-CNT) and ZnAC_2_–CNT (Zn-CNT) were used to describe carbon nanotubes impregnated with potassium (K) and zinc (Zn), as shown in Fig. 1. Raw carbon nanotubes are referred to as CNTs.

### Preparation of standard chromium(VI) solution

A stock solution of chromium(VI) (10 mg/L) was prepared by dissolving 0.028 g of potassium dichromate (Scharlau, Extra Pure) in deionized water (1000 mL). Following appropriate dilutions of the stock solution, working standard solutions were prepared.

### Preparation of reagent solutions

All the solutions were prepared from analytical reagent-grade chemicals and deionized water.

Potassium dichromate (Scharlau, Extra Pure) was prepared by dissolving 0.028 g of potassium dichromate in 1000 mL of deionized water to yield a 10 ppm solution. First, 1 M HCL–hydrochloric acid (Scharlau, 38% impurity) was prepared by dissolving 40.4 mL of HCl in 459.6 mL of deionized water to produce 1 M HCL acid. Over a period of one month, the solution concentration stabilized. NaOH (1 M) was prepared by mixing 8 g of NaOH with 200 mL of deionized water. When refrigerated, the solution was stable for one month. First, 0.5% 1,5-diphenyl carbazide (MERCK) was prepared by dissolving 5 g of DPC in acetone (1000 mL of acetone).

### Batch experimental procedures

A 1000 mL round-bottom flask was used to produce a 1 mg/L Cr(VI) solution. A total of 20 mL of this solution was transferred to various conical flasks. The solution was supplemented with CNTs, K-CNTs, AC-1, and AC-0 adsorbents. The mixture was then placed in a water bath shaker. The adsorbent solution was shaken at a constant speed and a constant temperature (25 °C).

The pH of the solution was adjusted using 1 M NaOH and 1 M HCl after it was prepared in a burette. Different experimental variables, including pH, contact time, concentration, and dose, were used in the experiment. To determine the effect of adsorbent dosage on Cr(VI) removal in a batch test, 24 clean conical flasks were obtained, washed, soaked in 1% H_2_SO_4_, rinsed, and dried in an oven.

Activated graphite powders (AC-0 and AC-1) and CNTs (K- and Zn-) at different dosages (5, 10, 20, 30, and 50 mg) were evaluated.

This was accomplished by adding the desired dose to a 20 mL solution of Cr(VI) and shaking it for 120 min at a constant agitation speed and a temperature of 25 °C. A batch test was conducted in 35 conical bottles to determine the influence of pH on Cr(VI) elimination. There were variations in the pH at 2, 3, 4, 6, 8, 10, and 12. After the adsorbents were added, the mixture was shaken at 25 °C for 120 min. Additionally, the pH of the Cr(VI) solution was adjusted to 2, and 20 mL samples were added to 24 conical flasks to examine the impact of time on Cr(VI) removal in the batch test.

The effect of time on the adsorbent was examined for all adsorbents at a constant agitation speed and at 25 °C (20, 40, 60, 80, 100, 120, 140, 160, 180, 200, and 240 min). The samples were subsequently collected for examination. At a constant agitation speed of 160 rpm, a temperature of 25 °C, and a 50 mg dosage, 20 mL of contaminated water was used, along with initial concentrations of 0.2, 1, 5, 10, 20, 30, and 40 mg/L, to examine the effects of altering the temperature initially. Furthermore, a regeneration batch test was performed at pH 8, and the adsorbents were recycled six times. Prior to reuse, every repurposed adsorbent was dried and cleaned with 0.3 M NaOH. Desorption occurred at 360-min intervals.

### Characterization of adsorbents and analytical procedure

The physical and chemical characteristics, surface morphology, and elemental composition of each adsorbent sample were studied by scanning electron microscopy (SEM S-4800, HITACHI, Japan). Additionally, the chemical and electronic states of the atoms in the adsorbent, along with their elemental composition, were determined via X-ray photoelectron spectroscopy (XPS, Thermo Fisher ESCALAB 250Xi, America). The stability and decay of the samples were observed via thermogravimetric analysis (TGA) at 20 mL/min, a temperature range of 25–600 °C, and a sample mass of 1 g. In addition, the chemical and electronic states of the atoms in the adsorbent, along with their elemental composition, were determined via X-ray photoelectron spectroscopy (XPS; Thermo Fisher ESCALAB 250Xi, USA).

A total of 100 µL of 1 M H_3_PO_4_ was added to 20 mL of the sample, followed by 400 µL of 0.5% DPC, and the mixture was incubated for at least 5 min before measurement via a UV‒visible spectrophotometer. The sample solutions were examined via the Cr(VI) calculation method. All the spectra and absorbance measurements were performed via a Jenway single-cell holder spectrophotometer with a 1.0 cm matched cell. pH was measured via a VIVOSUN digital pH meter. Briefly, 0.40 g of each adsorbent sample was added to 20 mL of distilled water and mechanically agitated for approximately 15 h. The pH of the solution was measured, and the experiment was performed at ambient temperature. In^38^, the absorption spectra of the colored products were scanned via a spectrophotometer in the wavelength range of 400–700 nm against the corresponding reagent blank. The optimal wavelength for maximum absorption was 540 nm, and the mixed reagents yielded similar findings; this mixture was used in this study.

### Regeneration

Regeneration was carried out under conditions very similar to those used in the analytical procedures, and the experiment was carried out at pH 8 for 30 min. The spent adsorbents were regenerated by using a 0.3 M NaOH solution after adsorption. The procedure was successful for functionalized and raw carbon nanotube (CNT) adsorbents. Desorption was performed at 6-h intervals.

## Results and discussion

### Characterization of adsorbents

**Fig. 2:**
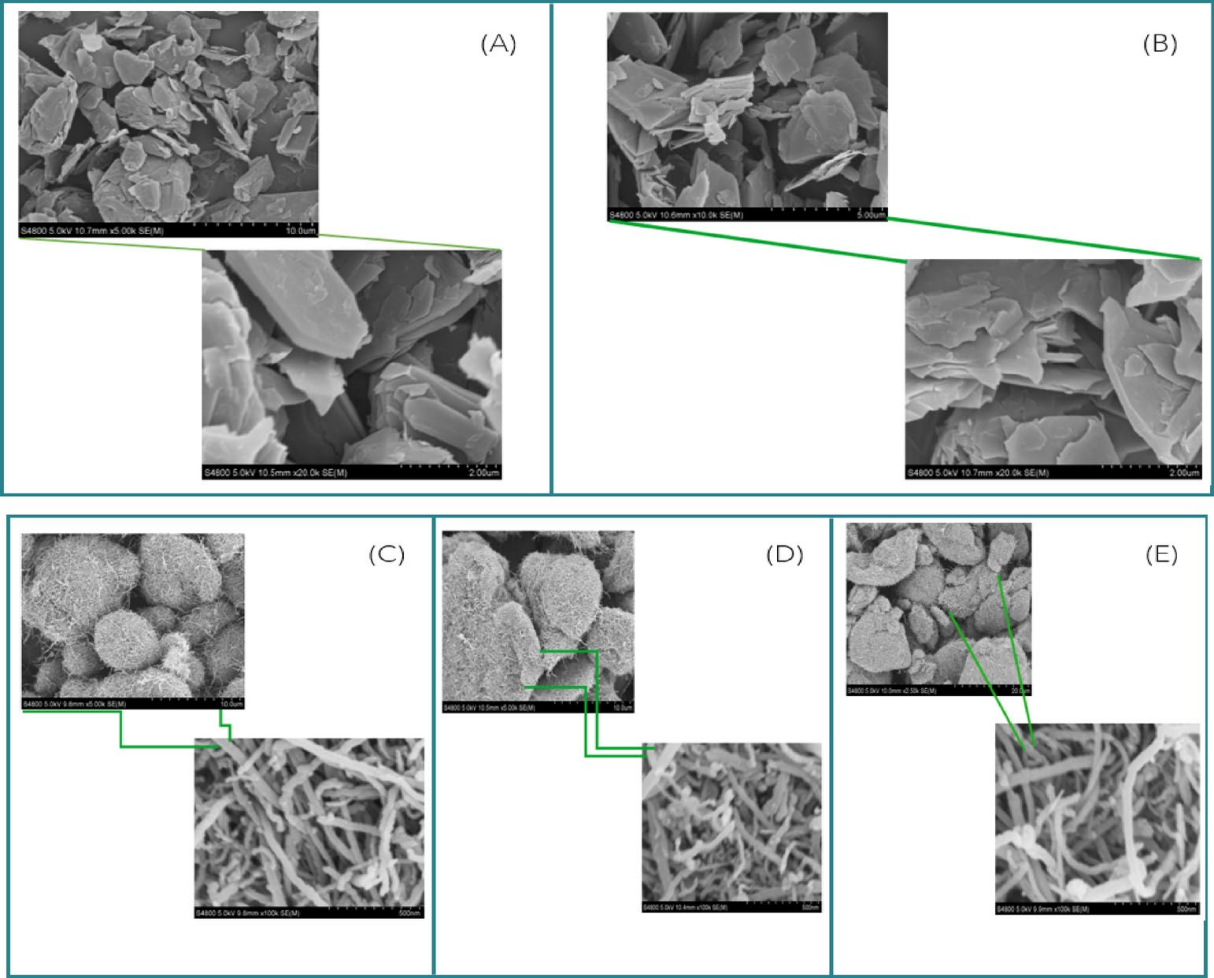
SEM images of (**a**) AC—0, Magnification: ×20,000. (**b**) AC—1, Magnification: ×20,000. (**c**) CNTs, Magnification: Left is ×5000 and Right is ×100,000. (**d**) K-CNTs, Magnification: Left is ×5000 and Right is ×100,000. (**e**) Zn—CNTs, Magnification: Left is ×2500 and Right is ×100,000.

As shown in Fig. 2, the change from spectra (A)–(B) to (C)–(E) represents a change in the synthesis or treatment process, for instance, from graphitization/exfoliation to catalytic carbon growth or activation. The creation of porous, fibrous networks in (C)–(E) is potentially useful in the fields of adsorption, catalysis, energy storage, and sensor materials owing to their high surface area and structural openness.

**Fig. 3:**
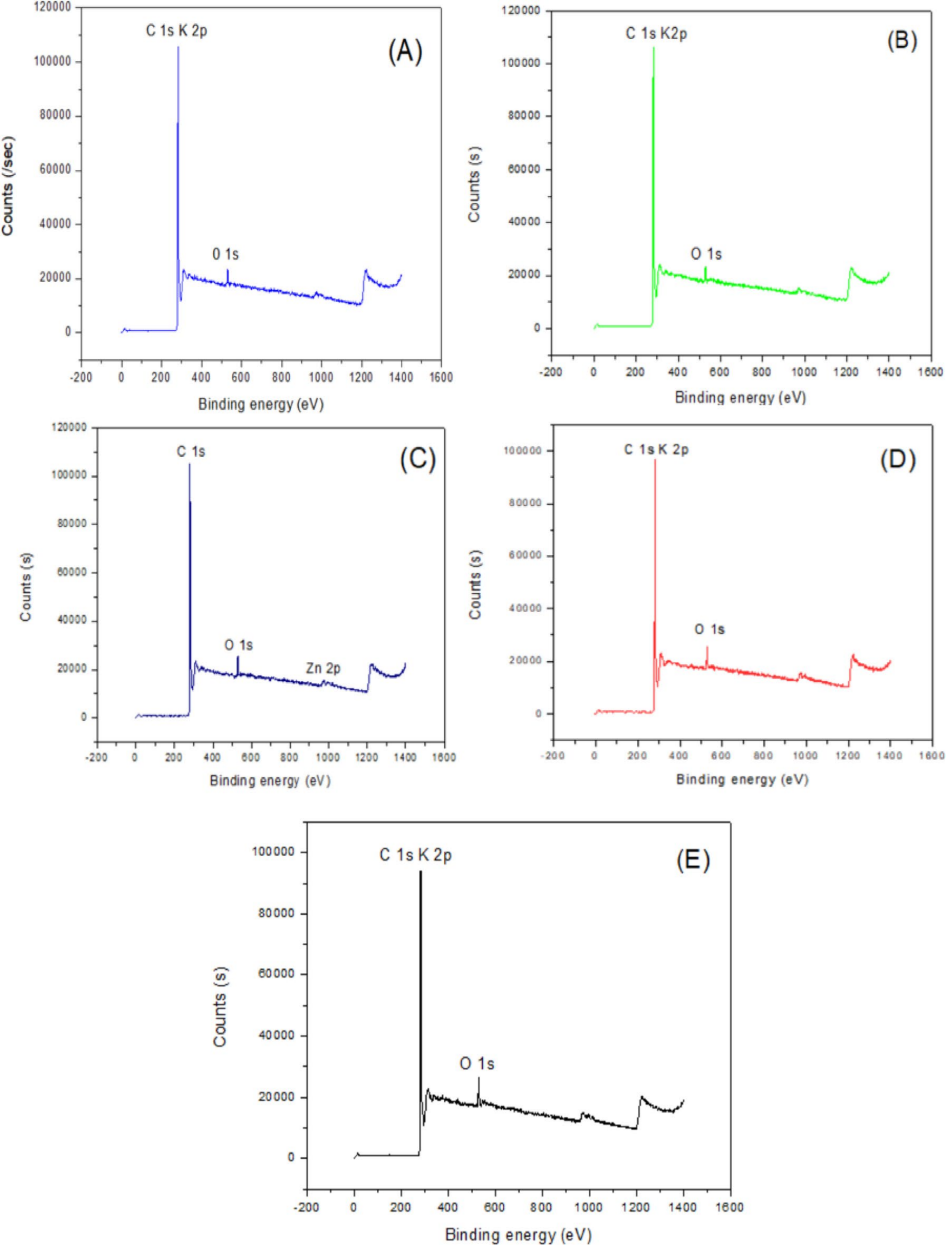
(**a**) XPS spectra of the CNT adsorbent, (**b**) K-CNT adsorbent, (**c**) Zn-CNT adsorbent, (**d**) AC-0 adsorbent, and (**e**) AC-1 adsorbent.

**Fig. 4:**
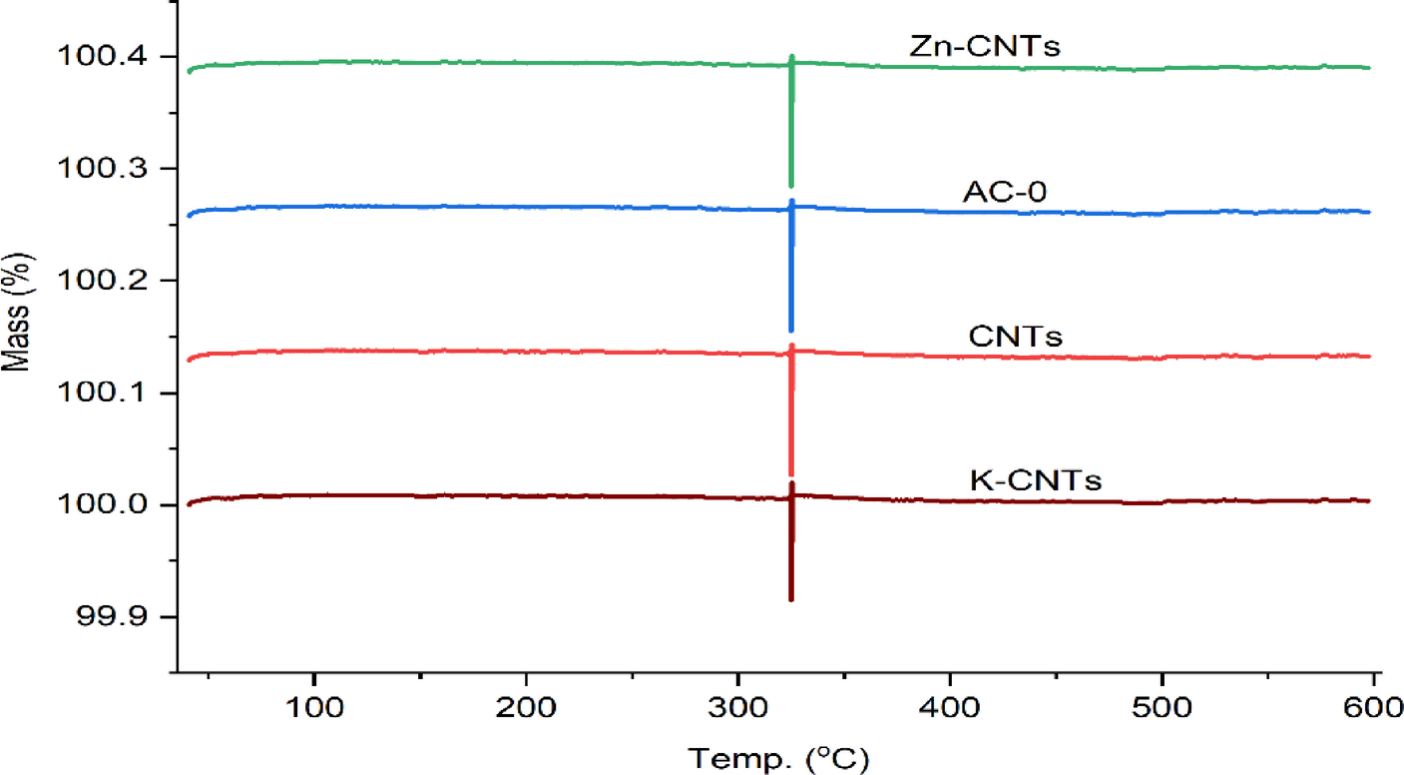
TGA thermograms of AC-0, CNTs, K-CNTs and Zn-CNTs.

The morphological differences also point toward probable elemental composition or functionalization differences, which is consistent with complementary analyses such as XPS analysis, as shown in Fig. 3. The XPS spectra collectively indicate that all the samples are carbonaceous but have undergone different surface treatments, as shown in Fig. 3. The presence of K and O signifies chemical activation and oxidation, and the Zn signal in the spectrum (C) signifies doping or surface loading with zinc species. These differences in surface chemistry can significantly influence the performance of the material in application fields such as adsorption, electrocatalysis, or energy storage. In Fig. 4, Zn-CNTs, AC-0, CNTs, and K-CNTs all sharply decrease or peak at approximately 300 °C, suggesting the occurrence of a common thermal event for all materials. These peaks indicate the temperature at which thermal degradation, weight loss, or oxidation occur, providing information on the thermal stability of each sample. The peak at approximately 300 °C suggested a significant thermal transition in all the samples, which could be due to desorption, decomposition, or oxidation reactions. The thermal stability above this temperature is high, as no significant weight loss is observed up to 600 °C.

### Effect of the adsorbent dosage

**Fig. 5:**
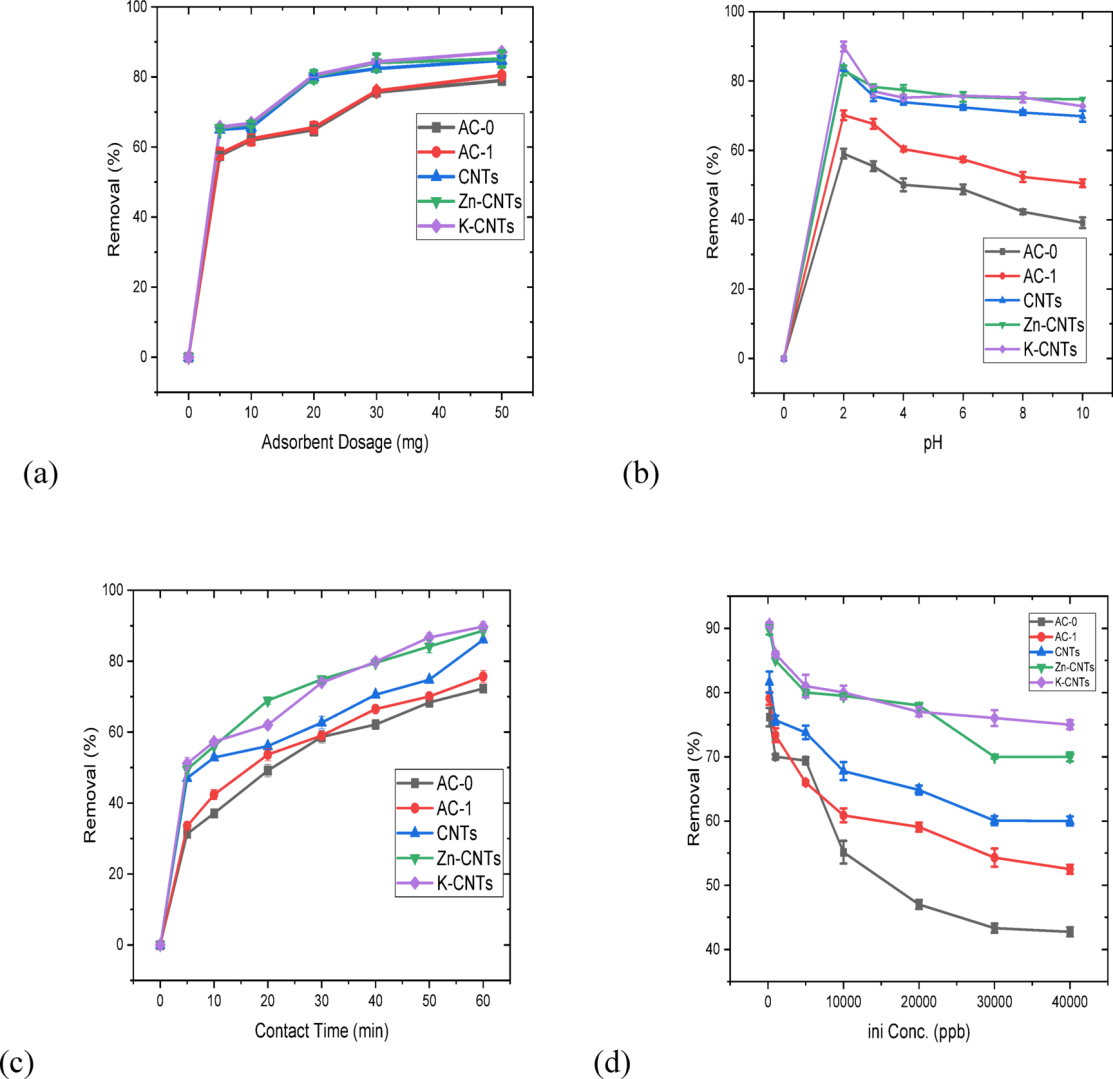
(**a**) Effect of adsorbent dosage on chromium removal. iniConc. of 1000 ppb/20 mL, a T of 25 °C, and a duration of 60 min. (**b**) The impact of pH variation on the complete removal of chromium. iniConc. of 1000 ppb, pH of 2, T of 25 °C, D of 50 mg/20 mL, time of 60 min. (**c**) Effect of contact duration on the residual chromium content. iniConc. of 1000 ppb/20 mL, a T of 25 °C, and a dosage of 50 mg. (**d**) Effect of varying the Cr(VI) concentration. Adsorption conditions: pHini = 2.0, [Dosage] of 50 mg/20 mL, temperature of 25 °C, time of 60 min.

CNTs were further explored in a 20 mL Cr(VI) solution with 5–50 mg of CNTs at pH 2 to analyze the influence of the component dosage on Cr(VI) elimination, as shown in Fig. 5a. The greatest improvement was obtained when the CNT concentration was 30 mg. The inclusion of functionalized CNTs clearly increased the removal efficiency, as shown in Fig. 5a. As the adsorbent dosage increased, more Cr(VI) was removed, and the removal efficiency continuously increased. With increasing mixture pH, the sorbent tends to deprotonate, and its adsorption capacity decreases. Thus, pH 2.0, pH 8 and pH 4.0 were used in all the subsequent assays. The CNT adsorbent had a maximal adsorption capacity 39.96 mg g^−1^ when the pH was 2.

The effects of various material doses on Cr(VI) elimination are shown in Fig. 5a. According to the data, 30 mg of CNTs can eliminate Cr(VI) in 2 h, whereas 30 mg of K-CNTs can eliminate Cr(VI) quickly in 3 min. Therefore, all subsequent tests were completed by adding 30 mg of K-, Zn-, CNTs, AC-1, or AC-0 to account for economic gains.

Similar observations were reported by^39^, in which granular ferric hydroxide adsorbents were used for chromium removal. A lower amount of adsorbent may achieve acceptable results because of the higher surface-area-to-volume ratio^39^. All the materials showed a sharp increase in removal at low adsorbent dosages. This means that at lower dosages, the available active sites account for contaminant removal. At dosages greater than 20 mg, the removal efficiency gradually increased and then plateaued. This means that above a certain dosage, an additional adsorbent does not play a significant role in removal. K-CNTs and Zn-CNTs exhibited maximum removal efficiencies at all dosages. They achieved 85–90% removal at high dosages. These CNTs are more efficient than activated carbon materials. The CNTs exhibited good performance, achieving up to 85% removal. The efficiency was lower than that of Zn-CNTs and K-CNTs but considerably greater than that of activated carbon. AC-1 is moderately efficient, achieving up to 80% removal at high dosages, which is superior to that of AC-0 but inferior to that of CNT-based materials. AC-0, which is the least effective material, removes an average of 70–75% of the material even when higher dosages are used, reflecting a lower adsorption capacity than those of the other materials. At low dosages, there are fewer active sites for adsorption, and hence, the removal efficiency is lower. As the dosage increased, more active sites were revealed, which contributed to a higher removal efficiency. With higher dosages, greater portions of contaminant molecules are already adsorbed, and the additional adsorbent contributes little extra effectiveness, so it plateaus. CNT-based materials are superior because of their larger surface area, which offers more adsorption sites. Zn-CNT and K-CNT surface modifications increase the adsorption efficiency. Activated carbons (AC-0 and AC-1) are inferior because they have a smaller surface area and fewer functional groups for effective adsorption.

### Effect of the solution pH

One of the main factors that determines the methods of metal ion adsorption is pH. The effect of pH on Cr(VI) adsorption is due to the interaction between ions in the mixture and the generated composite^40^. Cr(VI) (1 mg/L) was used as the initial concentration for the pH test. As the pH increased, less Cr(VI) was able to bind to the produced adsorbents.

Figure 5b showed that K-CNTs, Zn-CNTs, and CNTs were active in the acidic range, particularly at low pH values. At pH values greater than 8.0, the Cr(VI) adsorption performance tends to be low. The intensity of the chromium ions and the mixture pH strongly influence each complex, and various stable forms of Cr(VI), HCr_2_O^7−^, HCrO_4_^2−^, Cr_2_O_7_^2−^ and CrO_4_^2−^ are formed.

Figure 5b shows that the chromium RE decreased from 89 to 74% when the pH increased from 2.0 to 8.0 for K-CNTS. Chromium can be found in a variety of oxoanionic forms, including HCrO_4_ and Cr^2^O_7_^2+^, in acidic pH ranges (pH 2–6). CrO_4_^2+^ is the main ionic species above pH 6.0. The greater chromium RE at lower pH values can be attributed to H^+^ ions being absorbed by the SO^4−^ of the sulfate group. This may form complexes with Cr_2_O_7_^2+^ and HCrO^4−^ through electrostatic interactions^41^. The chromium RE decreases at higher pH values because of the competition between CrO_4_^2−^ and OH^−^ ions for the anion exchange sites in the adsorbent. A higher pH may cause the electron-rich polymer matrix to reduce Cr^6+^ to Cr^3+^, which would explain the removal of Cr^6+^^41^. A similar finding was reported when maghemite nanoparticles were used as adsorbents, where the chromium RE decreased with increasing pH, which was due to electrostatic contact between the chromium species and the adsorbent surface^41^.

Owing to protonation, the adsorbent is positively charged at lower pH values, whereas sorbates, such as dichromate ions, exist primarily as anions. This results in electrostatic allure between the sorbate and the sorbent, strengthening the adsorption at lower pH values.

### Effect of time on the adsorbent

The influence of exposure time on the depletion of Cr(VI) was explored at a pH of 2.0 for CNTs, Zn-CNTs, and graphitic carbons and at a pH of 4.0 for K-CNTS. The adsorbent concentration was set at 50 mg/20 mL for both carbon materials, and the solutions were equilibrated. An aliquot of the solution was taken periodically and examined via a Genway absorption spectrometer to determine Cr(VI) removal, and the results are displayed in Fig. 5c. All the materials initially exhibited a rapid increase in percent removal owing to the presence of active adsorption sites. The removal rate was highest during the initial 10–30 min. Beyond the initial period, the adsorption process became slower, indicating that the active sites were being occupied. At 60 min, the majority of the materials approach equilibrium, and no further appreciable increase in percentage removal is noted. K-CNTs had the highest removal efficiency at all times. Modification with potassium (K), which improves adsorption characteristics, results in a greater surface area or more functional groups to adsorb onto contaminants. Zn-CNTs constitute the second most common material, behind only K-CNTs. The presence of zinc (Zn) improved adsorption by increasing the number of active sites. CNTs are better than activated carbon materials (AC-0 and AC-1). In general, CNTs have a large surface area and good adsorption properties. The performance of AC-1 was better than that of AC-0 because of surface treatments. However, these materials remain less efficient than CNT-based materials. AC-0 has the lowest removal efficiency of all the materials. This is due to fewer active adsorption sites or less porosity than in other materials.

### Effect of varying the Cr(VI) concentration

The chromium (VI) adsorbent dose (50 g/20 mL), starting concentration (1–20 mg/L, pH 2 and 4.0), temperature (25 °C), and contact time (120 min) were fixed, and the percent adsorption of Cr(VI) was determined. As the initial chromium content increased, the percentage of chromium adsorbed decreased, as shown in Fig. 5d. At initial Cr(VI) concentrations less than or equal to 1 mg/L, the percentage removal was greater than 90%; however, as the concentration increased, the percentage removal steadily decreased. An increase in chromium content results in exhaustion of the adsorption sites. The starting metal concentration affects the equilibrium concentration, metal ion uptake rate, and kinetic characteristics. For all the materials, with increasing initial concentration, the removal efficiency decreases. This means that at higher concentrations, the adsorption sites become full and cannot be further removed. At lower initial concentrations, all the materials were removed excellently, with removal percentages above 70–80%. This implies that at lower contaminant levels, there are enough active sites to adsorb. K-CNTs had the best removal efficiency at all concentrations. Even at high levels, its efficiency is more than 70%, i.e., it has good adsorption capacity. Zn-CNTs are slightly less effective than K-CNTs, but they are very effective. The efficiency is relatively consistent even at high initial concentrations. The content of CNTs decreases moderately with increasing concentration.

Compared with activated carbon materials (AC-0 and AC-1), activated carbon materials (AC-0 and AC-1) exhibit improved performance but lower efficiency than modified CNTs (Zn-CNTs and K-CNTs). Compared with that of AC-0, the performance of AC-1 is better, but that of the CNT-based materials is lower. The percentage removal decreases significantly with increasing concentration, which indicates poor adsorption capacity. AC-0, which had the lowest performance, demonstrated a steep decrease in removal efficiency at elevated concentrations, indicating a weaker adsorption capacity, possibly because of fewer active sites and a smaller surface area. There are adequate active sites at low concentrations; hence, the removal efficiency is high. With increasing concentration, the number of active sites becomes saturated, and hence, the efficiency decreases. The competition for active sites is greater at higher concentrations of impurities, and it is challenging to achieve high percentage removal. There are more active sites, greater surface areas, and adsorption-enhancing groups on K-CNTs and Zn-CNTs. CNTs are effective because of their nanostructure but are inferior to functionalized CNTs. Activated carbon materials AC-0 and AC-1 are less effective because of their comparatively lower surface areas and fewer functional groups.

### Regeneration

**Fig. 6:**
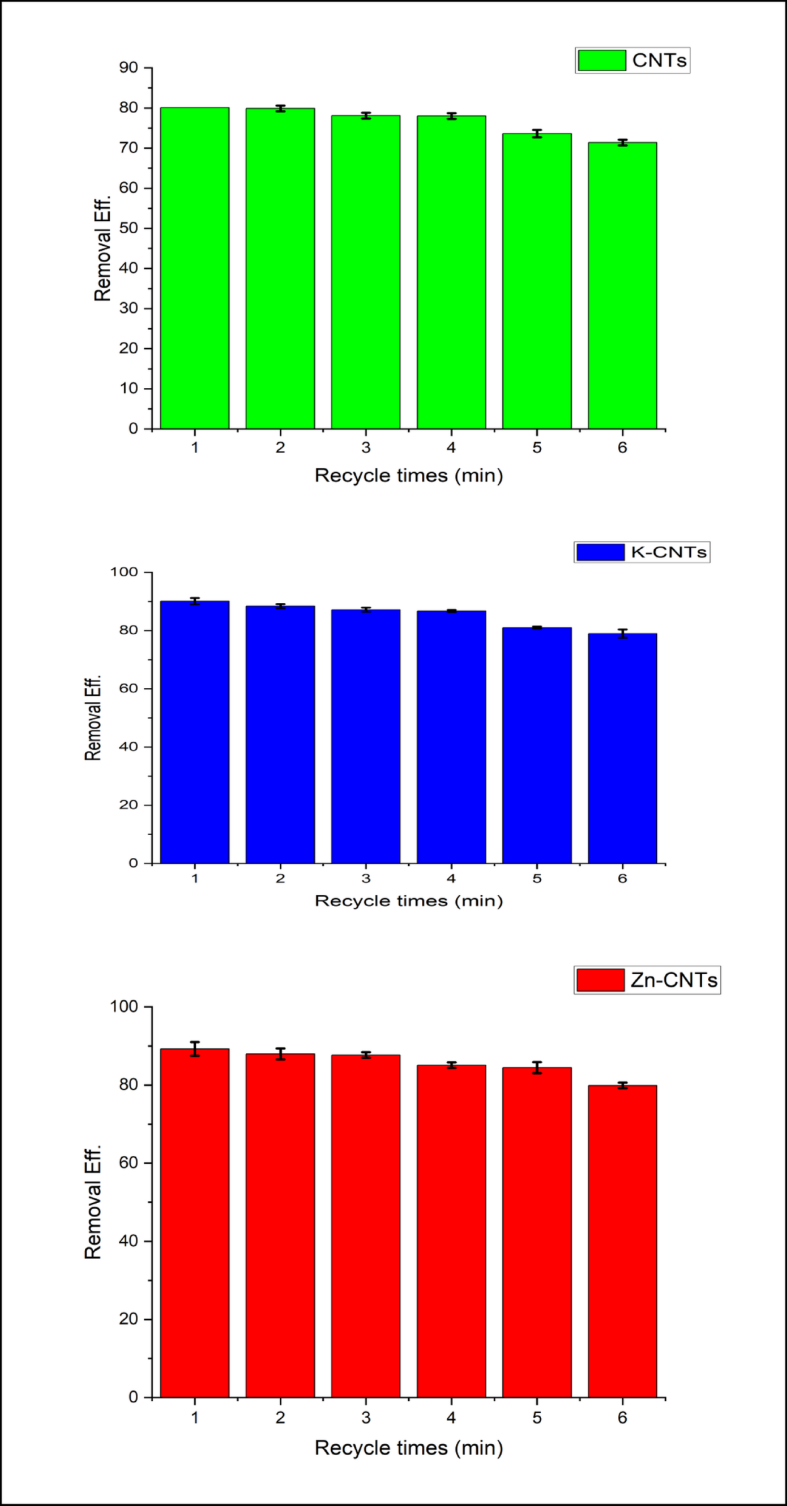
Regeneration of CNTs, K-CNTs and Zn-CNTs. Adsorption conditions: [Dosage] of 50 mg/20 mL, [ini Cr(VI)] of 1000 ppb, adsorption time of 30 min, and temperature of 25 °C. Desorption conditions: [Dosage] of 40 mg/mL, 0.3 M [NaOH], desorption time of 6 h, and temperature of 25 °C.

The Cr(VI) removal efficiency reached 95% and 32.7% after five adsorption/desorption cycle tests for K- and Zn-CNTs at neutral pH, i.e., pH = 8, and K-CNTs achieved 91% removal after 6 cycles, as shown in Fig. 6, which is particularly noteworthy. In contrast, the ability of the pure CNT adsorbent to remove Cr(VI) quickly decreased to 13% at pH = 8 after approximately two adsorption‒desorption cycles. The chemically grafted alkaline groups, which demonstrated great stability during the regeneration process, are responsible for the exceptional reusability of the functionalized CNT adsorbent.

### Adsorption kinetics

The kinetics of Cr(VI) adsorption onto pure CNTs and functionalized CNTs were explored using a constant agitation speed, initial Cr(VI) concentration of 200 ppb, and 10 mg of adsorbent at a temperature = 25 °C and time (1, 2, 3, 4, 5, 10, 20, and 30 min). The synthesized Zn‒CNT adsorbents achieved equilibrium 1 min earlier than did the pure CNT adsorbents. Owing to the high alkaline intensity on the Zn-CNT and K-CNT absorbent surfaces, it took just 3 min to achieve quick adsorption equilibrium.

**Fig. 7:**
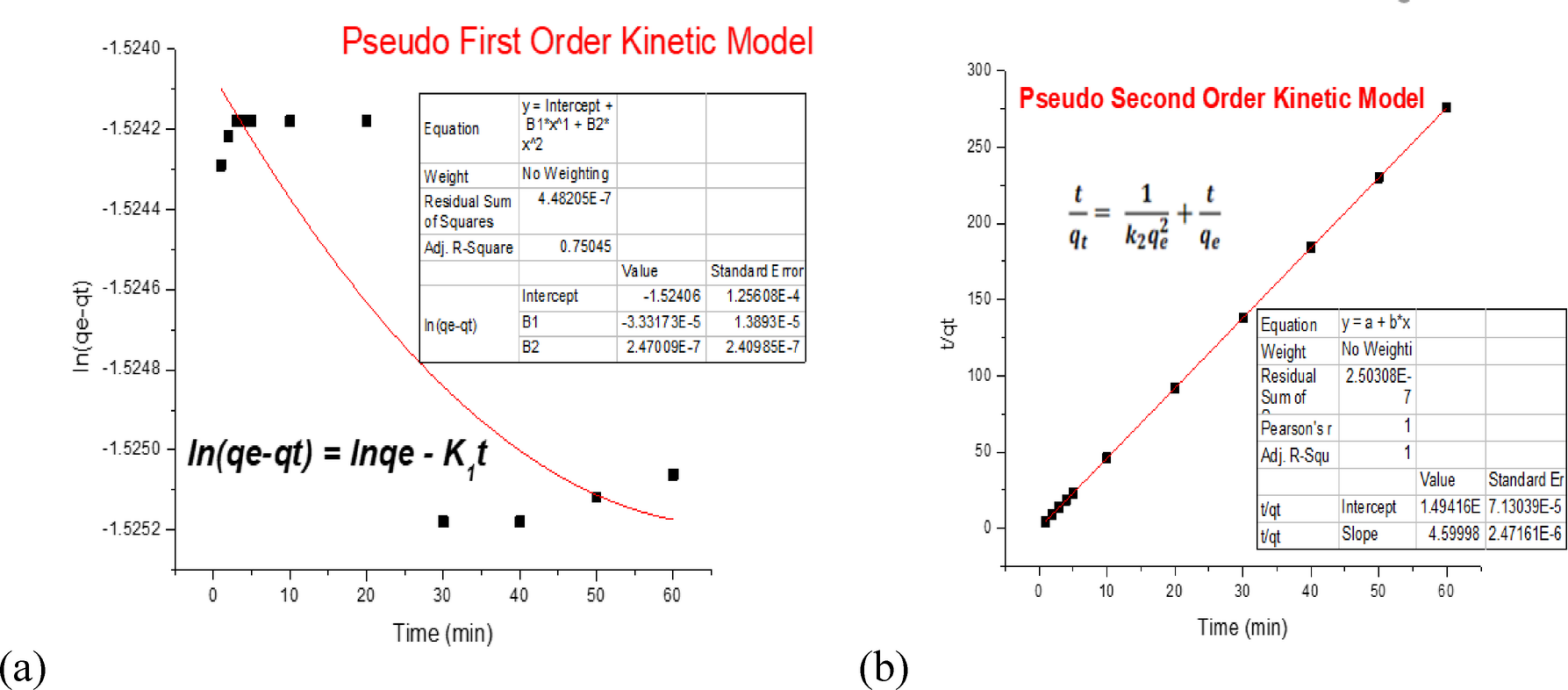
(**a**) Pseudo first-order kinetic model and (**b**) pseudo second-order kinetic model.

At 25 °C, 99% of the Cr(VI) was adsorbed in just 2 min at 200 ppb, and the K-CNTs demonstrated highly efficient adsorption potential, demonstrating the significant potential for the reduction of Cr(VI) from adulterated water, as shown in Fig. 7. However, as more Cr(VI) was adsorbed, the rate of Cr(VI) adsorption slowed. This can be attributed to the decrease in the number and gradient of active sites. Adsorption kinetics were explored to better understand the process via pseudo-first-order and pseudo-second-order kinetic models, as shown in Tables 1 and 2^42^. The two models can be represented via Eqs. 1 and 2 in their linearized forms.

The pseudofirst- and pseudo-second-order kinetic models are given by the following equations:

1 $${\text{ln }}\left[ {{\text{qe}}-{\text{ q}}\left( {\text{t}} \right)={\text{log}}\left( {{\text{qt}}} \right)} \right] - {{\text{k}}_{\text{1}}}\text{t} +{\text{lnqe}}$$

2 $$\frac{t}{{q\left( t \right)}}=\left( {\frac{1}{{{q_e}}}} \right)t + \frac{1}{{{k_2}q_{e}^{2}}}$$

In Fig. 7, the line does not pass through all the dots, and R^2^ is not 1; the first-order kinetic model is not suitable for the experimental data.

Since the line passes through all the dots, the R^2^ value is 1, and the values of qe from the model and qe from the experiment are close to each other; the pseudo-second-order kinetic model is suitable for the experimental data shown in Fig. 7.

### Adsorption isotherms

Adsorption isotherms were studied for Cr(VI) removal to describe how adsorbate molecules or ions interact with surface adsorption sites; correlation of the equilibrium data via a theoretical or empirical equation was used for interpretation and prediction of the adsorption rate^43^.

**Table 1:** Pseudo-first-order kinetic model interpretation.

Intercept	Slope	q_e_ (mg/g)	k_1_	*R* ^2^
− 1.52404	− 0.08145	7.59364E−06	0.0013575	0.75045

**Table 2:** Pseudo-second-order kinetic model interpretation.

Intercept	Slope	q_e_ (mg/g)	q_e_^2^	K^2^	*R* ^2^
1.49E−04	4.59998	0.21739225	0.16	4.18E+04	1

#### 1. Freundlich adsorption isotherm

Mathematically, the Freundlich adsorption isotherm can be defined in its linear form, as shown in Eq. (3).

3 $${\text{Log}}\;x/m ={\text{ logK }}+1/n\; {\text{log}}\;{\text{Ce}}$$

**Fig. 8:**
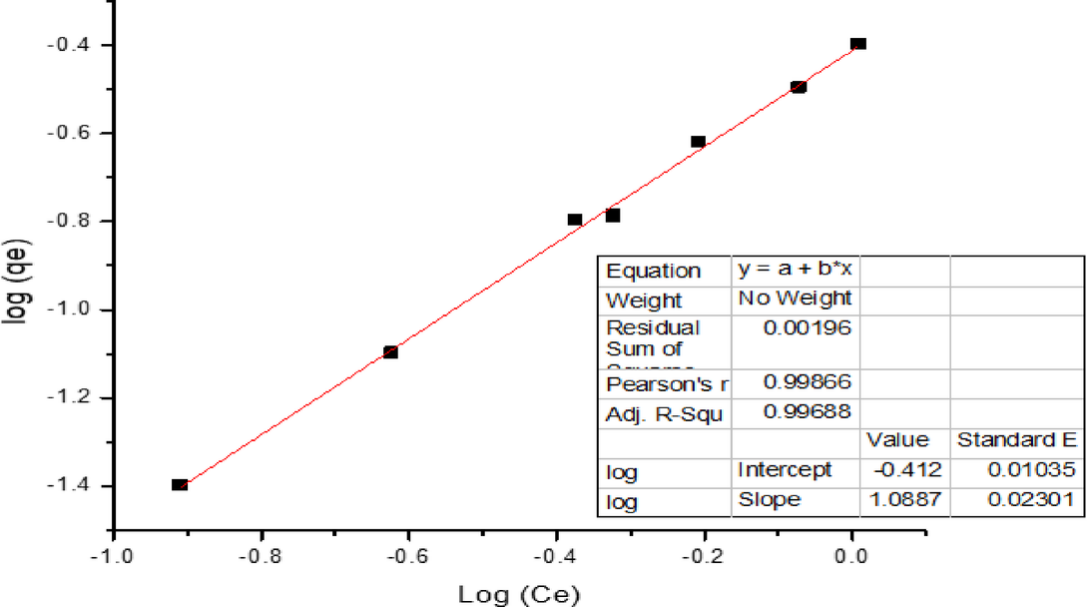
Freundlich adsorption isotherm.

Through the graphical analysis shown in Fig. 8, the adsorption capacity values for n and Kf (L mg^−1^) were analyzed. The results show that the adsorbent surface strongly favors Cr(VI) ions, as demonstrated by the sorption intensity value and the “n” value. Since 1/*n* is between 0 and 1 in Table 3 and follows the adsorption model, the experimental data are favorable for the Freundlich adsorption mechanism.

#### 2. Langmuir isotherm

Where K_L_ is the Langmuir constant and Ci is the initial concentration of the metal ion. The value of the separation factor R_L_ provides important information about the nature of adsorption. The value of R_L_ is between 0 and 1 for favorable adsorption, while R_L_ > 1 represents unfavorable adsorption, and R_L_ = 1 represents linear adsorption. The adsorption process is irreversible if R_L_ = 0.

**Fig. 9:**
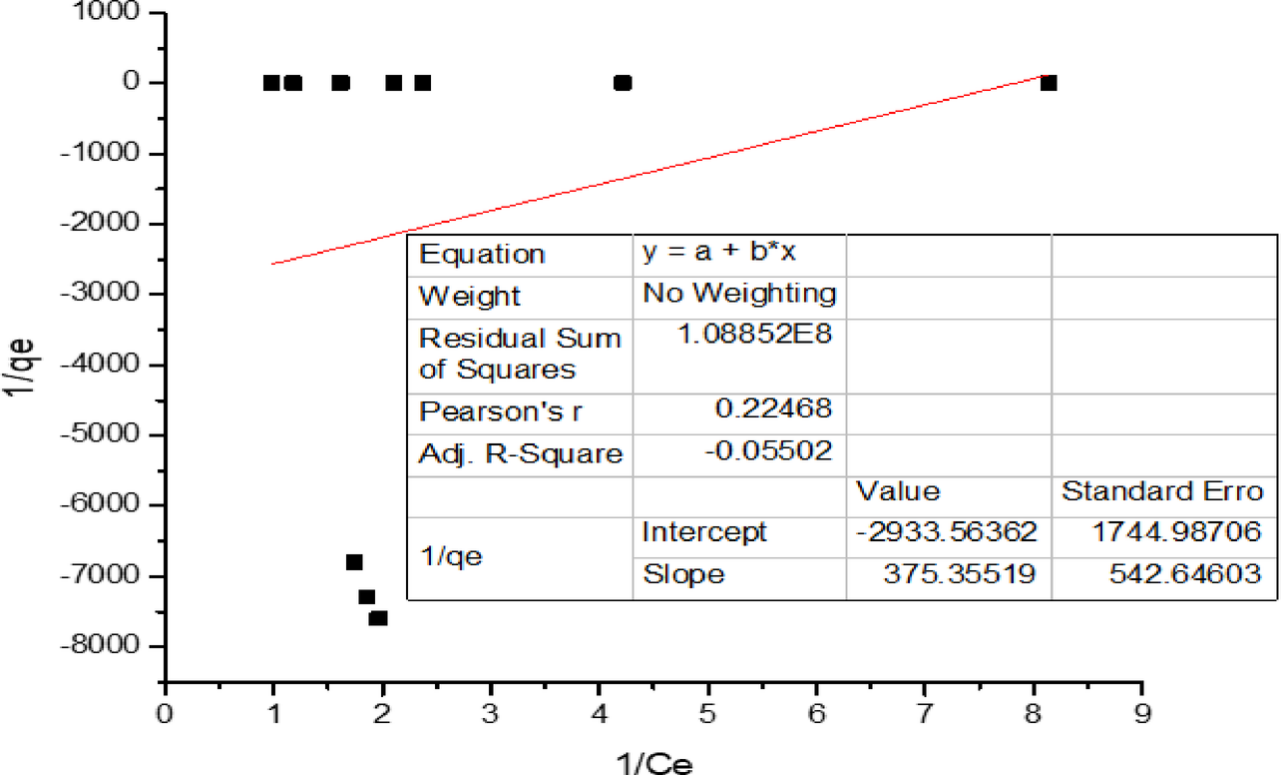
Langmuir isotherm plot.

Since the value of R_L_ is not between 0 and 1 in Table 3; Fig. 9, the experimental data do not favor the Langmuir adsorption isotherm.

#### 3. Dubinin–Radushkevich isotherm (D-R) model

**Fig. 10:**
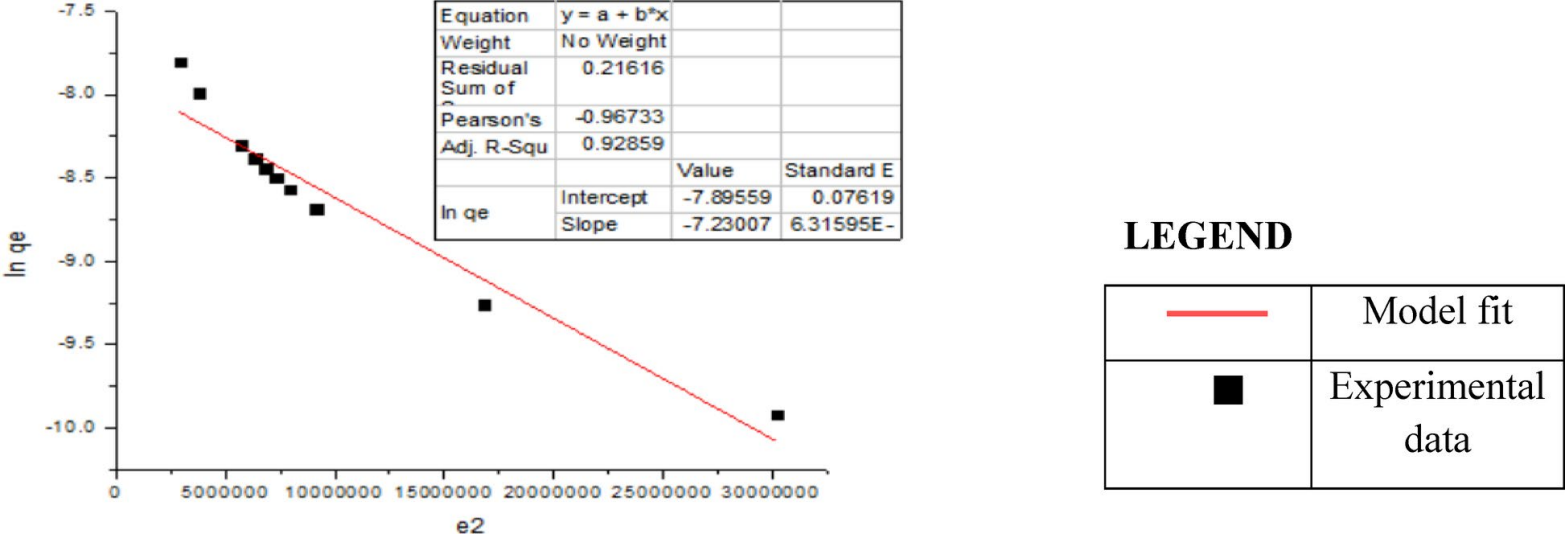
Dubinin–Radushkevich isotherm.

Where Q_D_ represents the theoretical maximum capacity, B_D_ represents the D‒R model constant, T represents the absolute temperature, K, and R represents the gas constant, kJ mol^−1^. The intercept and slope of the plot of ln (qe) versus ln (1 + 1/Ce) in Fig. 10 can be used to determine the values of Q_D_ and B_D_.

The mean energy of sorption, E (kJ mol^−1^), is determined via the following relationship:

4 $${\text{E}}={\text{1}}\sqrt {2{{\text{B}}_{\text{D}}}}$$

**Table 3:** Coefficients of two different sorption isotherm models for Cr(Vi) removal by carbon nanotubes and their correlation coefficients (R^2^. (Experimental conditions: dosage = 50 mg/20 mL; particle size = 100 nm; mixing rate = constant, T = 25 °C; contact time = 30 min for Cr(Vi); pH = 2.

	CNTs
Isotherm parameters	Cr (Vi)
Freundlish	
1/n	0.918526683
K_f_	0.394284768
R^2^	0.99882
Langmuir	
qmax (mg/g)	− 2.47408397
K_L_	− 0.3712593
R_L_	− 0.00270081
R^2^	− 0.05502
Dubinin–Radushkevich	
qm (mg/g)	0.66752
β (mol^2^/K^2^/J^2^)	1.09E+00
E (KJ/mol)	0.6776897
R^2^	0.9285

## Conclusion

It can be inferred from this work that functionalized CNT adsorbents can be produced affordably and broadly. Therefore, Cr(VI) was efficiently removed from aqueous solution at room pH. This substance demonstrated a comparatively high capacity for adsorbing chromium(VI) from aqueous solution.

Zn-CNTs and K-CNTs are the best materials and should be used preferably in applications requiring high adsorption efficiency. CNT-based materials outperform activated carbon; i.e., they can replace traditional adsorbents for better performance. Optimum contact time: As most of the adsorption occurs in the first 30 min, contact times longer than this may not considerably enhance the efficiency. K-CNTs and Zn-CNTs are the most effective adsorbents and have good removal efficiency across a range of concentrations. Traditional activated carbon materials (AC-0 and AC-1) are poor performers, especially at relatively high concentrations. These findings suggest that carbon nanotube-based materials are more appropriate for adsorption applications, especially when the concentration of contaminants is high.

K-CNTs and Zn-CNTs are the most effective adsorbents at different pH values and have high removal capacities even under neutral and basic pH conditions. Activated carbon materials (AC-0 and AC-1) are effective under acidic conditions but exhibit a sudden decrease in effectiveness at higher pH values. In conclusion, CNT-based materials are more suitable for adsorption applications where there is a change in pH. K-CNTs and Zn-CNTs are superior adsorbents, with better removal efficiency at low dosages. CNT-based materials are better than activated carbon and are thus ideal for adsorption processes. The results showed that an optimal dosage of 20–30 mg is ideal, as increasing the dosage beyond this dosage does not significantly influence removal.

This study demonstrated that, compared with activated carbon (AC-0 and AC-1), carbon nanotube materials (K-CNTs and Zn-CNTs in particular) exhibit greater adsorption efficiency. These materials qualify as good candidates for environmental or industrial use in decontamination.

A key limitation of the present study is that transmission electron microscopy (TEM) characterization of the adsorbents was not performed to obtain complete information on their nanoscale structure, morphology, or elemental composition. Whereas thermogravimetric analysis (TGA) measures mass changes (e.g., thermally stimulated changes), transmission electron microscopy (TEM) visually confirms the structural properties that give rise to such characteristics.

## Electronic supplementary material

Below is the link to the electronic supplementary material.

Supplementary Material 1

## Data Availability

All data generated or analysed during this study are included in this published article [and its supplementary information files].
