# Supplementary material for: Removal of hexavalent chromium from contaminated synthetic groundwater via functionalized carbon nanomaterials modified with zinc and potassium
Source: Sci Rep. 2025 Sep 30;15:33823. doi: 10.1038/s41598-025-01025-y (PMC12484932; doi:10.1038/s41598-025-01025-y)
Supplement: Supplementary file 1 — Supplementary Material 1 [file 41598_2025_1025_MOESM1_ESM.docx]

# **Appendix**

| **Journal:** | **Scientific Reports** |
| --- | --- |
| **Ref.:** | **8635eece-6cd8-447f-b80f-959eec3792a3** |
| **Title:** | **Removal of Hexavalent Chromium from Contaminated Synthetic Groundwater Using Functionalized Carbon Nanomaterials Modified with Zinc and Potassium** |
| **Authors:** | **P. D. Ibikunle, O.O. Elemile, P.O. Ejigboye, D.O. Bala, A. P. Olawolu, A.A. Adebayo and O.S. Olajide** |

Table A.1; **Effects of various concentrations**

|  |  |  | | **AC - 0** | |  | |  | | | |  |
| --- | --- | --- | --- | --- | --- | --- | --- | --- | --- | --- | --- | --- |
| **Initial Conc.** | **Conc. 1 (ppb)** | **Conc. 2 (ppb)** | | **Mean (ppb)** | | **Std.** | | **Removal Eff. %** | | | |  |
| 200.00 | 48.66 | 46.62 | | 47.64 | | 1.44 | | 76.18 | | | |  |
| 1000.00 | 299.50 | 300.00 | | 299.75 | | 0.35 | | 70.03 | | | |  |
| 5000.00 | 1530.50 | 1529.70 | | 1530.10 | | 0.57 | | 69.40 | | | |  |
| 10000.00 | 4484.50 | 4487.00 | | 4485.75 | | 1.77 | | 55.14 | | | |  |
| 20000.00 | 10601.00 | 10600.00 | | 10600.50 | | 0.71 | | 47.00 | | | |  |
| 30000.00 | 17004.50 | 17003.50 | | 17004.00 | | 0.71 | | 43.32 | | | |  |
| 40000.00 | 22901.00 | 22900.00 | | 22900.50 | | 0.71 | | 42.75 | | | |  |
|  |  |  | |  | |  | |  | | | |  |
| pH = 2 | Dosage = 50 mg | | | Time = 30 min | | | | Temp. = 25 °C | | | |  |
|  |  |  | **AC - 1** | | | |  | | |  | | |
| **Initial Conc.** | **Conc. 1 (ppb)** | **Conc. 2 (ppb)** | **Mean (ppb)** | | | | **Std.** | | | **Removal Eff. %** | | |
| 200.00 | 41.12 | 42.50 | 41.81 | | | | 0.98 | | | 79.10 | | |
| 1000.00 | 265.50 | 267.00 | 266.25 | | | | 1.06 | | | 73.38 | | |
| 5000.00 | 1700.50 | 1701.00 | 1700.75 | | | | 0.35 | | | 65.99 | | |
| 10000.00 | 3912.50 | 3911.00 | 3911.75 | | | | 1.06 | | | 60.88 | | |
| 20000.00 | 8187.00 | 8186.00 | 8186.50 | | | | 0.71 | | | 59.07 | | |
| 30000.00 | 13708.50 | 13710.50 | 13709.50 | | | | 1.41 | | | 54.30 | | |
| 40000.00 | 19000.00 | 19001.00 | 19000.50 | | | | 0.71 | | | 52.50 | | |
|  |  |  |  | | | |  | | |  | | |
| pH = 2 | Dosage = 50 mg | | Time = 2 hr | | | |  | | | Temp. = 25 °C | | |
|  | **CNTs** | | | |  | | | |  | |  |  |
| **Initial Conc.** | **Conc. 1 (ppb)** | **Conc. 2 (ppb)** | **Mean (ppb)** | | **Std.** | | | | **Removal Eff. %** | |  |  |
| 200.00 | 37.90 | 35.56 | 36.73 | | 1.65 | | | | 81.64 | |  |  |
| 1000.00 | 242.50 | 243.50 | 243.00 | | 0.71 | | | | 75.70 | |  |  |
| 5000.00 | 1310.00 | 1311.50 | 1310.75 | | 1.06 | | | | 73.79 | |  |  |
| 10000.00 | 3224.00 | 3222.00 | 3223.00 | | 1.41 | | | | 67.77 | |  |  |
| 20000.00 | 7032.00 | 7033.00 | 7032.50 | | 0.71 | | | | 64.84 | |  |  |
| 30000.00 | 11984.50 | 11983.50 | 11984.00 | | 0.71 | | | | 60.05 | |  |  |
| 40000.00 | 16000.50 | 15999.50 | 16000.00 | | 0.71 | | | | 60.00 | |  |  |
|  |  |  |  | |  | | | |  | |  |  |
| Dosage = 50 mg | | pH= 2 | Time = 30 min | | | | | | Temp. = 25 ^o^C | |  |  |

|  | |  | |  | | **Zn - CNTs** | |  | |  | | |
| --- | --- | --- | --- | --- | --- | --- | --- | --- | --- | --- | --- | --- |
| **Initial Conc.** | | **Conc. 1 (ppb)** | | **Conc. 2 (ppb)** | | **Mean (ppb)** | | **Std.** | | **Removal Eff. %** | | |
| 200.00 | | 20.00 | | 21.00 | | 20.50 | | 0.71 | | 89.75 | | |
| 1000.00 | | 150.00 | | 150.00 | | 150.00 | | 0.00 | | 85.00 | | |
| 5000.00 | | 1000.00 | | 999.50 | | 999.75 | | 0.35 | | 80.01 | | |
| 10000.00 | | 2050.00 | | 2049.60 | | 2049.80 | | 0.28 | | 79.50 | | |
| 20000.00 | | 4400.50 | | 4400.20 | | 4400.35 | | 0.21 | | 78.00 | | |
| 30000.00 | | 9000.87 | | 9000.49 | | 9000.68 | | 0.27 | | 70.00 | | |
| 40000.00 | | 12000.50 | | 12001.50 | | 12001.00 | | 0.71 | | 70.00 | | |
|  | |  | |  | |  | |  | |  | | |
| Dosage = 50 mg | |  | | pH= 2 | | Time = 30 min | |  | | Temp. = 25 ^o^C | | |
|  |  | |  | | **K - CNTs** | |  | |  | |  |  |
| **Initial Conc.** | **Conc. 1 (ppb)** | | **Conc. 2 (ppb)** | | **Mean (ppb)** | | **Std.** | | **Removal Eff. %** | | |  |
| 200.00 | 18.50 | | 19.00 | | 18.75 | | 0.35 | | 90.63 | |  |  |
| 1000.00 | 140.00 | | 139.50 | | 139.75 | | 0.35 | | 86.03 | |  |  |
| 5000.00 | 950.50 | | 948.00 | | 949.25 | | 1.77 | | 81.02 | |  |  |
| 10000.00 | 1997.00 | | 1998.50 | | 1997.75 | | 1.06 | | 80.02 | |  |  |
| 20000.00 | 4600.00 | | 4601.00 | | 4600.50 | | 0.71 | | 77.00 | |  |  |
| 30000.00 | 7188.89 | | 7190.65 | | 7189.77 | | 1.24 | | 76.03 | |  |  |
| 40000.00 | 10000.00 | | 9999.00 | | 9999.50 | | 0.71 | | 75.00 | |  |  |
|  |  | |  | |  | |  | |  | |  |  |
| pH = 4 | Dosage = 50 mg | |  | | Time = 30 min | | | | Temp. = 25 ^o^C | | |  |

Table A.2**: Effects of time on the adsorbent**

|  |  | | | | | **AC - 0** | | | | |  | | | | |  | | | |  | | | |  | | | |  | | | |
| --- | --- | --- | --- | --- | --- | --- | --- | --- | --- | --- | --- | --- | --- | --- | --- | --- | --- | --- | --- | --- | --- | --- | --- | --- | --- | --- | --- | --- | --- | --- | --- |
| **Time (min)** | **Conc. 1 (ppb)** | | | | | **Conc. 2 (ppb)** | | | | | **Mean (ppb)** | | | | | **Std.** | | | | **Removal Eff. %** | | | | | | | |  | | | |
| **0.00** | **0.00** | | | | | **0.00** | | | | | **0.00** | | | | | **0.00** | | | | **0.00** | | | |  | | | |  | | | |
| 5.00 | 687.00 | | | | | 686.50 | | | | | 686.75 | | | | | 0.35 | | | | 31.33 | | | |  | | | |  | | | |
| 10.00 | 628.00 | | | | | 629.50 | | | | | 628.75 | | | | | 1.06 | | | | 37.13 | | | |  | | | |  | | | |
| 20.00 | 509.50 | | | | | 507.00 | | | | | 508.25 | | | | | 1.77 | | | | 49.18 | | | |  | | | |  | | | |
| 30.00 | 414.50 | | | | | 412.00 | | | | | 413.25 | | | | | 1.77 | | | | 58.68 | | | |  | | | |  | | | |
| 40.00 | 379.50 | | | | | 377.50 | | | | | 378.50 | | | | | 1.41 | | | | 62.15 | | | |  | | | |  | | | |
| 50.00 | 316.52 | | | | | 315.70 | | | | | 316.11 | | | | | 0.58 | | | | 68.39 | | | |  | | | |  | | | |
| 60.00 | 277.00 | | | | | 277.00 | | | | | 277.00 | | | | | 0.00 | | | | 72.30 | | | |  | | | |  | | | |
|  |  | | | | |  | | | | |  | | | | |  | | | |  | | | |  | | | |  | | | |
| **Initial Cr VI Conc. = 1000 ppb** | | | | | | **pH = 2** | | | | | **Temp. = 25 °C** | | | | | | | | | **Shaking speed =???** | | | | | | | | **Dosage = 50 mg** | | | |
|  | | **AC - 1** | | | | | | | |  | | | |  | | | |  | | | | |  | | |  | | |  |  |  |
| **Time (min)** | | **Conc. 1 (ppb)** | | | **Conc. 2 (ppb)** | | | | | **Mean (ppb)** | | | | **Std.** | | | | **Removal Eff. %** | | | | | | | |  | | |  |  |  |
| **0.00** | | **0.00** | | | **0.00** | | | | | **0.00** | | | | **0.00** | | | | **0.00** | | | | |  | | |  | | |  |  |  |
| 5.00 | | 664.50 | | | 664.50 | | | | | 664.50 | | | | 0.00 | | | | 33.55 | | | | |  | | |  | | |  |  |  |
| 10.00 | | 575.00 | | | 577.00 | | | | | 576.00 | | | | 1.41 | | | | 42.40 | | | | |  | | |  | | |  |  |  |
| 20.00 | | 462.00 | | | 464.50 | | | | | 463.25 | | | | 1.77 | | | | 53.68 | | | | |  | | |  | | |  |  |  |
| 30.00 | | 410.00 | | | 409.50 | | | | | 409.75 | | | | 0.35 | | | | 59.03 | | | | |  | | |  | | |  |  |  |
| 40.00 | | 334.50 | | | 335.50 | | | | | 335.00 | | | | 0.71 | | | | 66.50 | | | | |  | | |  | | |  |  |  |
| 50.00 | | 299.00 | | | 300.00 | | | | | 299.50 | | | | 0.71 | | | | 70.05 | | | | |  | | |  | | |  |  |  |
| 60.00 | | 242.00 | | | 244.50 | | | | | 243.25 | | | | 1.77 | | | | 75.68 | | | | |  | | |  | | |  |  |  |
|  | |  | | |  | | | | |  | | | |  | | | |  | | | | |  | | |  | | |  |  |  |
| **Initial Cr VI Conc. = 1000 ppb** | | | | | **pH = 2** | | | | | **Temp. = 25 °C** | | | | | | | | **Shaking speed =???** | | | | | | | | **Dosage = 50 mg** | | |  |  |  |
|  | | | | | **CNTs** | | | | |  | | | | | | | |  | | | | | | | |  | | |  |  |  |
|  | |  | |  | | | | |  | | | | | |  | | | | | |  | | | | | |  | | | |  |
| **Time (min)** | | **Conc. 1 (ppb)** | | **Conc. 2 (ppb)** | | | | | **Mean (ppb)** | | | | | | **Std.** | | | | | | **Removal Eff. %** | | | | | | | | | |  |
| **0.00** | | **0.00** | | **0.00** | | | | | **0.00** | | | | | | **0.00** | | | | | | **0.00** | | | | | |  | | | |  |
| 5.00 | | 529.50 | | 529.50 | | | | | 529.50 | | | | | | 0.00 | | | | | | 47.05 | | | | | |  | | | |  |
| 10.00 | | 471.00 | | 472.00 | | | | | 471.50 | | | | | | 0.71 | | | | | | 52.85 | | | | | |  | | | |  |
| 20.00 | | 439.50 | | 439.00 | | | | | 439.25 | | | | | | 0.35 | | | | | | 56.08 | | | | | |  | | | |  |
| 30.00 | | 372.00 | | 374.50 | | | | | 373.25 | | | | | | 1.77 | | | | | | 62.68 | | | | | |  | | | |  |
| 40.00 | | 294.20 | | 294.50 | | | | | 294.35 | | | | | | 0.21 | | | | | | 70.57 | | | | | |  | | | |  |
| 50.00 | | 252.00 | | 252.00 | | | | | 252.00 | | | | | | 0.00 | | | | | | 74.80 | | | | | |  | | | |  |
| 60.00 | | 139.50 | | 139.50 | | | | | 139.50 | | | | | | 0.00 | | | | | | 86.05 | | | | | |  | | | |  |
|  | |  | |  | | | | |  | | | | | |  | | | | | |  | | | | | |  | | | |  |
| Initial Cr VI Conc. = 1000 ppb | | | | pH = 2 | | | | | Temp. = 25 °C | | | | | | Dosage = 50 mg | | | | | | Shaking speed =??? | | | | | | | | | |  |
|  | | |  | | | | |  | | | | | **Zn - CNTs** | | | | | |  | | | | | |  | | | |  |  |  |
| **Time (min)** | | | **Conc. 1 (ppb)** | | | | | **Conc. 2 (ppb)** | | | | | **Mean (ppb)** | | | | | | **Std.** | | | | | | **Removal Eff. %** | | | |  |  |  |
| **0.00** | | | 0.00 | | | | | 0.00 | | | | | 0.00 | | | | | | 0.00 | | | | | | 0.00 | | | |  |  |  |
| 5.00 | | | 506.00 | | | | | 504.50 | | | | | 505.25 | | | | | | 1.06 | | | | | | 49.48 | | | |  |  |  |
| 10.00 | | | 439.50 | | | | | 439.50 | | | | | 439.50 | | | | | | 0.00 | | | | | | 56.05 | | | |  |  |  |
| 20.00 | | | 311.50 | | | | | 310.00 | | | | | 310.75 | | | | | | 1.06 | | | | | | 68.93 | | | |  |  |  |
| 30.00 | | | 250.00 | | | | | 251.50 | | | | | 250.75 | | | | | | 1.06 | | | | | | 74.93 | | | |  |  |  |
| 40.00 | | | 205.00 | | | | | 204.50 | | | | | 204.75 | | | | | | 0.35 | | | | | | 79.53 | | | |  |  |  |
| 50.00 | | | 159.50 | | | | | 157.00 | | | | | 158.25 | | | | | | 1.77 | | | | | | 84.18 | | | |  |  |  |
| 60.00 | | | 114.17 | | | | | 113.97 | | | | | 114.07 | | | | | | 0.14 | | | | | | 88.59 | | | |  |  |  |
|  | | |  | | | | |  | | | | |  | | | | | |  | | | | | |  | | | |  |  |  |
| Initial Cr VI Conc. = 1000 ppb | | | | | | | | pH = 2 | | | | | Temp. = 25 °C | | | | | | Dosage = 50 mg | | | | | | Shaking speed =??? | | | |  |  |  |
|  | | | | | | | |  | | | | | K - CNTs | | | | | |  | | | | | |  | | | |  |  |  |
| **Time (min)** | **Conc. 1 (ppb)** | | | | | | **Conc. 2 (ppb)** | | | | | **Mean (ppb)** | | | | | **Std.** | | | | | **Removal Eff. %** | | | | | | | |  |  |
| 0.00 | 0.00 | | | | | | 0.00 | | | | | 0.00 | | | | | 0.00 | | | | | 0.00 | | | | | | | |  |  |
| **5.00** | 487.00 | | | | | | 489.50 | | | | | 488.25 | | | | | 1.77 | | | | | 51.18 | | | | | | | |  |  |
| 10.00 | 427.00 | | | | | | 427.00 | | | | | 427.00 | | | | | 0.00 | | | | | 57.30 | | | | | | | |  |  |
| 20.00 | 380.50 | | | | | | 379.00 | | | | | 379.75 | | | | | 1.06 | | | | | 62.03 | | | | | | | |  |  |
| 30.00 | 261.00 | | | | | | 260.00 | | | | | 260.50 | | | | | 0.71 | | | | | 73.95 | | | | | | | |  |  |
| 40.00 | 202.00 | | | | | | 200.50 | | | | | 201.25 | | | | | 1.06 | | | | | 79.88 | | | | | | | |  |  |
| 50.00 | 132.00 | | | | | | 134.00 | | | | | 133.00 | | | | | 1.41 | | | | | 86.70 | | | | | | | |  |  |
| 60.00 | 101.50 | | | | | | 103.50 | | | | | 102.50 | | | | | 1.41 | | | | | 89.75 | | | | | | | |  |  |
|  |  | | | | | |  | | | | |  | | | | |  | | | | |  | | | | | | | |  |  |
| **Initial Cr VI Conc. = 1000 ppb** | | | | | | | **pH = 4** | | | | | **Temp. = 25 °C** | | | | | **Dosage = 50 mg** | | | | | **Shaking speed =???** | | | | | | | |  |  |

Table A.3: **Effect of pH on the adsorbent**

|  | | **AC-0** | | | | | | | | |  | | | |  | | | | |  |  |  |
| --- | --- | --- | --- | --- | --- | --- | --- | --- | --- | --- | --- | --- | --- | --- | --- | --- | --- | --- | --- | --- | --- | --- |
| **pH** | | **Conc. 1 (ppb)** | | | **Conc. 2 (ppb)** | | | **Mean (ppb)** | | | **Std.** | | | | **Removal Eff. %** | | | | |  |  |  |
| **0.00** | | **0.00** | | | **0.00** | | | **0.00** | | | **0.00** | | | | **0.00** | | | | |  |  |  |
| 2.00 | | 408.60 | | | 410.60 | | | 409.60 | | | 1.41 | | | | 59.04 | | | | |  |  |  |
| 3.00 | | 446.60 | | | 444.60 | | | 445.60 | | | 1.41 | | | | 55.44 | | | | |  |  |  |
| 4.00 | | 498.00 | | | 500.60 | | | 499.30 | | | 1.84 | | | | 50.07 | | | | |  |  |  |
| 6.00 | | 511.60 | | | 513.60 | | | 512.60 | | | 1.41 | | | | 48.74 | | | | |  |  |  |
| 8.00 | | 576.60 | | | 577.60 | | | 577.10 | | | 0.71 | | | | 42.29 | | | | |  |  |  |
| 10.00 | | 607.40 | | | 609.60 | | | 608.50 | | | 1.56 | | | | 39.15 | | | | |  |  |  |
|  | |  | | |  | | |  | | |  | | | |  | | | | |  |  |  |
|  | |  | | |  | | |  | | |  | | | |  | | | | |  |  |  |
| **Initial Cr VI Conc. = 1000 ppb** | | | | | **pH = 2** | | | **Temp. = 25 °C** | | | | | | | **Shaking speed =???** | | | | |  |  |  |
|  | |  | | |  | | |  | | |  | | | |  | | | | |  |  |  |
|  | | | **AC-1** | | | | | | | | | |  | | | |  | | | | |  |
| **pH** | | | **Conc. 1 (ppb)** | | **Conc. 2 (ppb)** | | **Mean (ppb)** | | | | | | **Std.** | | | | **Removal Eff. %** | | | | |  |
| **0.00** | | | **0.00** | | **0.00** | | **0.00** | | | | | | **0.00** | | | | **0.00** | | | | |  |
| 2.00 | | | 299.60 | | 297.60 | | 298.60 | | | | | | 1.41 | | | | 70.14 | | | | |  |
| 3.00 | | | 324.60 | | 322.60 | | 323.60 | | | | | | 1.41 | | | | 67.64 | | | | |  |
| 4.00 | | | 395.60 | | 396.60 | | 396.10 | | | | | | 0.71 | | | | 60.39 | | | | |  |
| 6.00 | | | 425.60 | | 426.60 | | 426.10 | | | | | | 0.71 | | | | 57.39 | | | | |  |
| 8.00 | | | 477.60 | | 475.60 | | 476.60 | | | | | | 1.41 | | | | 52.34 | | | | |  |
| 10.00 | | | 494.00 | | 495.60 | | 494.80 | | | | | | 1.13 | | | | 50.52 | | | | |  |
|  | | |  | |  | |  | | | | | |  | | | |  | | | | |  |
|  | | |  | |  | |  | | | | | |  | | | |  | | | | |  |
| **Initial Cr VI Conc. = 1000 ppb** | | | | | **pH = 2** | | **Temp. = 25 °C** | | | | | |  | | | | **Shaking speed =???** | | | | |  |
|  | | | **CNTs** | | | | | | | | | |  | | | | |  | | | | |
| **pH** | | | **Conc. 1 (ppb)** | | **Conc. 2 (ppb)** | | **Mean (ppb)** | | | | | | **Std.** | | | | | **Removal Eff. %** | | | | |
| **0.00** | | | **0.00** | | **0.00** | | **0.00** | | | | | | **0.00** | | | | | **0.00** | | | | |
| 2.00 | | | 163.60 | | 162.60 | | 163.10 | | | | | | 0.71 | | | | | 83.69 | | | | |
| 3.00 | | | 244.60 | | 242.60 | | 243.60 | | | | | | 1.41 | | | | | 75.64 | | | | |
| 4.00 | | | 260.60 | | 261.60 | | 261.10 | | | | | | 0.71 | | | | | 73.89 | | | | |
| 6.00 | | | 275.60 | | 276.60 | | 276.10 | | | | | | 0.71 | | | | | 72.39 | | | | |
| 8.00 | | | 290.60 | | 291.60 | | 291.10 | | | | | | 0.71 | | | | | 70.89 | | | | |
| 10.00 | | | 300.40 | | 302.60 | | 301.50 | | | | | | 1.56 | | | | | 69.85 | | | | |
|  | | |  | |  | |  | | | | | |  | | | | |  | | | | |
|  | | |  | |  | |  | | | | | |  | | | | |  | | | | |
| **Initial Cr VI Conc. = 1000 ppb** | | | | | **pH = 2** | | **Temp. = 25 °C** | | | | | |  | | | | | **Shaking speed =???** | | | | |
|  | **Zn-CNTs** | | | | | |  | | |  | | | |  | | | | |  |  |  |  |
| **pH** | **Conc. 1 (ppb)** | | | | **Conc. 2 (ppb)** | | **Mean (ppb)** | | | **Std.** | | | | **Removal Eff. %** | | | | |  |  |  |  |
| **0.00** | **0.00** | | | | **0.00** | | **0.00** | | | **0.00** | | | | **0.00** | | | | |  |  |  |  |
| 2.00 | 170.60 | | | | 168.60 | | 169.60 | | | 1.41 | | | | 83.04 | | | | |  |  |  |  |
| 3.00 | 217.60 | | | | 216.60 | | 217.10 | | | 0.71 | | | | 78.29 | | | | |  |  |  |  |
| 4.00 | 224.60 | | | | 226.60 | | 225.60 | | | 1.41 | | | | 77.44 | | | | |  |  |  |  |
| 6.00 | 244.60 | | | | 246.60 | | 245.60 | | | 1.41 | | | | 75.44 | | | | |  |  |  |  |
| 8.00 | 250.60 | | | | 250.60 | | 250.60 | | | 0.00 | | | | 74.94 | | | | |  |  |  |  |
| 10.00 | 252.60 | | | | 252.60 | | 252.60 | | | 0.00 | | | | 74.74 | | | | |  |  |  |  |
|  |  | | | |  | |  | | |  | | | |  | | | | |  |  |  |  |
|  |  | | | |  | |  | | |  | | | |  | | | | |  |  |  |  |
| **Initial Cr VI Conc. = 1000 ppb** | | | | | **pH = 2** | | **Temp. = 25 °C** | | | | | | | **Shaking speed =???** | | | | |  |  |  |  |
|  | | | | **K-CNTs** | | | | |  | | |  | | | |  | | | | |  |  |
| **pH** | | | | **Conc. 1 (ppb)** | | **Conc. 2 (ppb)** | | | **Mean (ppb)** | | | **Std.** | | | | **Removal Eff. %** | | | | |  |  |
| **0.00** | | | | **0.00** | | **0.00** | | | **0.00** | | | **0.00** | | | | **0.00** | | | | |  |  |
| 2.00 | | | | 101.60 | | 99.60 | | | 100.60 | | | 1.41 | | | | 89.94 | | | | |  |  |
| 3.00 | | | | 230.60 | | 228.60 | | | 229.60 | | | 1.41 | | | | 77.04 | | | | |  |  |
| 4.00 | | | | 248.60 | | 247.60 | | | 248.10 | | | 0.71 | | | | 75.19 | | | | |  |  |
| 6.00 | | | | 242.60 | | 242.60 | | | 242.60 | | | 0.00 | | | | 75.74 | | | | |  |  |
| 8.00 | | | | 248.60 | | 246.60 | | | 247.60 | | | 1.41 | | | | 75.24 | | | | |  |  |
| 10.00 | | | | 272.60 | | 272.60 | | | 272.60 | | | 0.00 | | | | 72.74 | | | | |  |  |
|  | | | |  | |  | | |  | | |  | | | |  | | | | |  |  |
|  | | | |  | |  | | |  | | |  | | | |  | | | | |  |  |
| **Initial Cr VI Conc. = 1000 ppb** | | | | | | **pH = 2** | | | **Temp. = 25 °C** | | | | | | | **Shaking speed =???** | | | | |  |  |

Table A.4: **Effects of adsorbent dosage**

|  | | | |  | | | | | | **AC-0** | | | | | | | | | | | | | | |  | | |
| --- | --- | --- | --- | --- | --- | --- | --- | --- | --- | --- | --- | --- | --- | --- | --- | --- | --- | --- | --- | --- | --- | --- | --- | --- | --- | --- | --- |
| Adsorbent Dosage (mg) | | | | Conc. Ppb | | | | | | Duplicate | | | | | | Removal % | | | | | | Mean | | | Std | | |
| **0.00** | | | | **0.00** | | | | | | **0.00** | | | | | | **0.00** | | | | | | **0.00** | | | **0.00** | | |
| 5.00 | | | | 423.67 | | | | | | 425.00 | | | | | | 57.57 | | | | | | 424.34 | | | 0.94 | | |
| 10.00 | | | | 381.65 | | | | | | 380.00 | | | | | | 61.92 | | | | | | 380.83 | | | 1.17 | | |
| 20.00 | | | | 349.65 | | | | | | 351.65 | | | | | | 64.94 | | | | | | 350.65 | | | 1.41 | | |
| 30.00 | | | | 244.00 | | | | | | 243.35 | | | | | | 75.63 | | | | | | 243.68 | | | 0.46 | | |
| 50.00 | | | | 210.00 | | | | | | 210.00 | | | | | | 79.00 | | | | | | 210.00 | | | 0.00 | | |
|  | | | |  | | | | | |  | | | | | |  | | | | | |  | | |  | | |
|  | | | |  | | | | | |  | | | | | |  | | | | | |  | | |  | | |
|  | | | |  | | | | | |  | | | | | |  | | | | | |  | | |  | | |
| Time = 2 hr | | | | | | | | | |  | | | | | |  | | | | | |  | | |  | | |
| Initial Conc. = 1000 ppb | | | | | | | | | | | | | | | |  | | | | | |  | | |  | | |
| pH = 3 | | | |  | | | | | |  | | | | | |  | | | | | |  | | |  | | |
| Temp. = 25 °C | | | | | | | | | |  | | | | | |  | | | | | |  | | |  | | |
| Shaking Speed = | | | | | | | | | |  | | | | | |  | | | | | |  | | |  | | |
|  | | |  | | | | | **AC-1** | | | | | | |  | | | | |  | | | |  | | |  |
| Adsorbent Dosage (mg) | | | Conc. Ppb | | | | | Duplicate | | | | | | | Removal % | | | | | Mean | | | | Std | | |  |
| **0.00** | | | **0.00** | | | | | **0.00** | | | | | | | **0.00** | | | | | **0.00** | | | | **0.00** | | |  |
| 5.00 | | | 420.00 | | | | | 418.00 | | | | | | | 58.10 | | | | | 419.00 | | | | 1.41 | | |  |
| 10.00 | | | 377.67 | | | | | 375.33 | | | | | | | 62.35 | | | | | 376.50 | | | | 1.65 | | |  |
| 20.00 | | | 345.33 | | | | | 343.33 | | | | | | | 65.57 | | | | | 344.33 | | | | 1.41 | | |  |
| 30.00 | | | 240.33 | | | | | 239.63 | | | | | | | 76.00 | | | | | 239.98 | | | | 0.49 | | |  |
| 50.00 | | | 195.67 | | | | | 194.89 | | | | | | | 80.47 | | | | | 195.28 | | | | 0.55 | | |  |
|  | | |  | | | | |  | | | | | | |  | | | | |  | | | |  | | |  |
|  | | |  | | | | |  | | | | | | |  | | | | |  | | | |  | | |  |
|  | | |  | | | | |  | | | | | | |  | | | | |  | | | |  | | |  |
| Time = 2 hr | | | | | | | |  | | | | | | |  | | | | |  | | | |  | | |  |
| Initial Conc. = 200 ppb | | | | | | | | | | | | | | |  | | | | |  | | | |  | | |  |
| pH = 3 | | |  | | | | |  | | | | | | |  | | | | |  | | | |  | | |  |
| Temp. = 25 °C | | | | | | | |  | | | | | | |  | | | | |  | | | |  | | |  |
| Shaking Speed = | | | | | | | |  | | | | | | |  | | | | |  | | | |  | | |  |
|  | | | | |  | | | | **CNTs** | | | | |  | | | |  | | | | |  | | |  |  |
| Adsorbent Dosage (mg) | | | | | Conc. Ppb | | | | Duplicate | | | | | Removal % | | | | Mean | | | | | Std | | |  |  |
| **0.00** | | | | | **0.00** | | | | **0.00** | | | | | **0.00** | | | | **0.00** | | | | | **0.00** | | |  |  |
| 5.00 | | | | | 350.00 | | | | 350.01 | | | | | 65.00 | | | | 350.01 | | | | | 0.01 | | |  |  |
| 10.00 | | | | | 343.35 | | | | 344.94 | | | | | 65.59 | | | | 344.15 | | | | | 1.12 | | |  |  |
| 20.00 | | | | | 201.65 | | | | 200.65 | | | | | 79.89 | | | | 201.15 | | | | | 0.71 | | |  |  |
| 30.00 | | | | | 175.79 | | | | 176.65 | | | | | 82.38 | | | | 176.22 | | | | | 0.61 | | |  |  |
| 50.00 | | | | | 151.95 | | | | 153.35 | | | | | 84.74 | | | | 152.65 | | | | | 0.99 | | |  |  |
|  | | | | |  | | | |  | | | | |  | | | |  | | | | |  | | |  |  |
|  | | | | |  | | | |  | | | | |  | | | |  | | | | |  | | |  |  |
|  | | | | |  | | | |  | | | | |  | | | |  | | | | |  | | |  |  |
| Time = 2 hr | | | | |  | | | |  | | | | |  | | | |  | | | | |  | | |  |  |
| Initial Conc. = 1000 ppb | | | | | | | | |  | | | | |  | | | |  | | | | |  | | |  |  |
| pH = 3 | | | | |  | | | |  | | | | |  | | | |  | | | | |  | | |  |  |
| Temp. = 25 °C | | | | |  | | | |  | | | | |  | | | |  | | | | |  | | |  |  |
| Shaking Speed = | | | | | | | | |  | | | | |  | | | |  | | | | |  | | |  |  |
|  | |  | | | | | **Zn-CNTs** | | | | |  | | |  | |  | | | |  |  |  |  |  |  |  |
| Adsorbent Dosage (mg) | | Conc. Ppb | | | | | Duplicate | | | | | Removal % | | | Mean | | Std | | | |  |  |  |  |  |  |  |
| **0.00** | | **0.00** | | | | | **0.00** | | | | | **0.00** | | | **0.00** | | **0.00** | | | |  |  |  |  |  |  |  |
| 5.00 | | 345.62 | | | | | 346.51 | | | | | 65.39 | | | 346.07 | | 0.63 | | | |  |  |  |  |  |  |  |
| 10.00 | | 334.39 | | | | | 333.44 | | | | | 66.61 | | | 333.92 | | 0.67 | | | |  |  |  |  |  |  |  |
| 20.00 | | 197.36 | | | | | 199.69 | | | | | 80.15 | | | 198.53 | | 1.65 | | | |  |  |  |  |  |  |  |
| 30.00 | | 157.28 | | | | | 160.72 | | | | | 84.10 | | | 159.00 | | 2.43 | | | |  |  |  |  |  |  |  |
| 50.00 | | 146.94 | | | | | 150.19 | | | | | 85.14 | | | 148.57 | | 2.30 | | | |  |  |  |  |  |  |  |
|  | |  | | | | |  | | | | |  | | |  | |  | | | |  |  |  |  |  |  |  |
|  | |  | | | | |  | | | | |  | | |  | |  | | | |  |  |  |  |  |  |  |
|  | |  | | | | |  | | | | |  | | |  | |  | | | |  |  |  |  |  |  |  |
| Time = 2 hr | | | | | | |  | | | | |  | | |  | |  | | | |  |  |  |  |  |  |  |
| Initial Conc. = 1000 ppb | | | | | | | | | | | |  | | |  | |  | | | |  |  |  |  |  |  |  |
| pH = 3 | |  | | | | |  | | | | |  | | |  | |  | | | |  |  |  |  |  |  |  |
| Temp. = 25 °C | | | | | | |  | | | | |  | | |  | |  | | | |  |  |  |  |  |  |  |
| Shaking Speed = | | | | | | |  | | | | |  | | |  | |  | | | |  |  |  |  |  |  |  |
|  |  | | | | | **K-CNTs** | | | | |  | |  | | | | | |  | | | | |  |  |  |  |
| Adsorbent Dosage (mg) | Conc. Ppb | | | | | Duplicate | | | | | Removal % | | Mean | | | | | | Std | | | | |  |  |  |  |
| **0.00** | **0.00** | | | | | **0.00** | | | | | **0.00** | | **0.00** | | | | | | **0.00** | | | | |  |  |  |  |
| 5.00 | 343.23 | | | | | 342.68 | | | | | 65.70 | | 342.96 | | | | | | 0.39 | | | | |  |  |  |  |
| 10.00 | 330.91 | | | | | 332.43 | | | | | 66.83 | | 331.67 | | | | | | 1.07 | | | | |  |  |  |  |
| 20.00 | 195.68 | | | | | 193.61 | | | | | 80.54 | | 194.65 | | | | | | 1.46 | | | | |  |  |  |  |
| 30.00 | 155.44 | | | | | 157.87 | | | | | 84.33 | | 156.66 | | | | | | 1.72 | | | | |  |  |  |  |
| 50.00 | 129.61 | | | | | 128.73 | | | | | 87.08 | | 129.17 | | | | | | 0.62 | | | | |  |  |  |  |
|  |  | | | | |  | | | | |  | |  | | | | | |  | | | | |  |  |  |  |
|  |  | | | | |  | | | | |  | |  | | | | | |  | | | | |  |  |  |  |
|  |  | | | | |  | | | | |  | |  | | | | | |  | | | | |  |  |  |  |
| Time = 2 hr | | | | | |  | | | | |  | |  | | | | | |  | | | | |  |  |  |  |
| Initial Conc. = 1000 ppb | | | | | | | | | | |  | |  | | | | | |  | | | | |  |  |  |  |
| pH = 3 |  | | | | |  | | | | |  | |  | | | | | |  | | | | |  |  |  |  |
| Temp. = 25 °C | | | | | |  | | | | |  | |  | | | | | |  | | | | |  |  |  |  |
| Shaking Speed = | | | | | |  | | | | |  | |  | | | | | |  | | | | |  |  |  |  |

Table A.5; **Regeneration**

|  | **CNTs** | | | |  | | |  | | | |  | | |  |  |
| --- | --- | --- | --- | --- | --- | --- | --- | --- | --- | --- | --- | --- | --- | --- | --- | --- |
| **Conc. 1** | **Conc. 2** | **Mean (ppb)** | | | **Std.** | | | **Removal Eff. %** | | | | **Recycle times** | | |  |  |
| 199.00 | 199.00 | 199.00 | | | 0.00 | | | 80.10 | | | | 1.00 | | |  |  |
| 200.50 | 201.50 | 201.00 | | | 0.71 | | | 79.90 | | | | 2.00 | | |  |  |
| 218.50 | 219.50 | 219.00 | | | 0.71 | | | 78.10 | | | | 3.00 | | |  |  |
| 219.50 | 220.50 | 220.00 | | | 0.71 | | | 78.00 | | | | 4.00 | | |  |  |
| 263.00 | 264.30 | 263.65 | | | 0.92 | | | 73.64 | | | | 5.00 | | |  |  |
| 286.50 | 285.50 | 286.00 | | | 0.71 | | | 71.40 | | | | 6.00 | | |  |  |
|  |  |  | | |  | | |  | | | |  | | |  |  |
| pH = 4 | Dosage: 50 mg | | | | Time: 30 min | | |  | | | | Initial Conc. = 1000 ppb | | |  |  |
|  | | | **K - CNTs** | | |  | | |  | |  | | |  | | |
| **Conc. 1** | | | **Conc. 2** | | | **Mean (ppb)** | | | **Std.** | | **Removal Eff. %** | | | **Recycle times** | | |
| 98.00 | | | 99.50 | | | 98.75 | | | 1.06 | | 90.13 | | | 1.00 | | |
| 115.50 | | | 116.50 | | | 116.00 | | | 0.71 | | 88.40 | | | 2.00 | | |
| 127.50 | | | 128.50 | | | 128.00 | | | 0.71 | | 87.20 | | | 3.00 | | |
| 133.05 | | | 132.50 | | | 132.78 | | | 0.39 | | 86.72 | | | 4.00 | | |
| 190.00 | | | 189.50 | | | 189.75 | | | 0.35 | | 81.03 | | | 5.00 | | |
| 209.50 | | | 211.50 | | | 210.50 | | | 1.41 | | 78.95 | | | 6.00 | | |
|  | | |  | | |  | | |  | |  | | |  | | |
| pH = 4 | | | Dosage: 50 mg | | |  | | | Time: 30 min | |  | | | Initial Conc. = 1000 ppb | | |
|  | **Zn - CNTs** | |  |  | | |  | | |  | | |  | | |  |
| **Conc. 1** | **Conc. 2** | | **Mean (ppb)** | **Std.** | | | **Removal Eff. %** | | | **Recycle times** | | | | | |  |
| 106.00 | 108.50 | | 107.25 | 1.77 | | | 89.28 | | | 1.00 | | |  | | |  |
| 121.50 | 119.50 | | 120.50 | 1.41 | | | 87.95 | | | 2.00 | | |  | | |  |
| 122.50 | 123.50 | | 123.00 | 0.71 | | | 87.70 | | | 3.00 | | |  | | |  |
| 149.50 | 148.50 | | 149.00 | 0.71 | | | 85.10 | | | 4.00 | | |  | | |  |
| 156.50 | 154.50 | | 155.50 | 1.41 | | | 84.45 | | | 5.00 | | |  | | |  |
| 200.50 | 201.50 | | 201.00 | 0.71 | | | 79.90 | | | 6.00 | | |  | | |  |
|  |  | |  |  | | |  | | |  | | |  | | |  |
|  | pH = 4 | | Dosage: 50 mg | | | | Time: 30 min | | |  | | | Initial Conc. = 1000 ppb | | |  |

| Time(min) | Ci(mg/L) | Ce(mg/L) | qt (mg/g) | t/qt |
| --- | --- | --- | --- | --- |
| 1 | 544.5 | 1.08 | 0.217368 | 4.600493 |
| 2 | 544.5 | 1.04 | 0.217384 | 9.200309 |
| 3 | 544.5 | 1.02 | 0.217392 | 13.79996 |
| 4 | 544.5 | 1.02 | 0.217392 | 18.39994 |
| 5 | 544.5 | 1.02 | 0.217392 | 22.99993 |
| 10 | 544.5 | 1.02 | 0.217392 | 45.99985 |
| 20 | 544.5 | 1.02 | 0.217392 | 91.99971 |
| 30 | 544.5 | 1.02 | 0.217392 | 137.9996 |
| 40 | 544.5 | 1.02 | 0.217392 | 183.9994 |
| 50 | 544.5 | 1.02 | 0.217392 | 229.9993 |
| 60 | 544.5 | 1.02 | 0.217392 | 275.9991 |
